# Plasma redox imbalance caused by albumin oxidation promotes lung-predominant NETosis and pulmonary cancer metastasis

**DOI:** 10.1038/s41467-018-07550-x

**Published:** 2018-11-30

**Authors:** Minoru Inoue, Ryota Nakashima, Masahiro Enomoto, Yuhki Koike, Xiao Zhao, Kenneth Yip, Shao Hui Huang, John N. Waldron, Mitsuhiko Ikura, Fei-Fei Liu, Scott V. Bratman

**Affiliations:** 1 Princess Margaret Cancer Centre Research Institute, University Health Network, Toronto, M5G2M9 ON Canada; 2 Department of Gastrointestinal and Pediatric Surgery, Mie University Graduate School of Medicine, Tsu City, 514-8507 Mie Prefecture Japan; 3 Radiation Medicine Program, Princess Margaret Cancer Centre, University Healthy Network, Toronto, M5G2M9 ON Canada; 4 Department of Radiation Oncology, University of Toronto, Toronto, M5G2M9 ON Canada; 5 Department of Medical Biophysics, University of Toronto, Toronto, M5G2M9 ON Canada

## Abstract

Neutrophil extracellular traps (NETs) promote cancer metastasis in preclinical models following massive exogenous inflammatory stimuli. It remains unknown whether cancer hosts under physiologic conditions experience NETosis and consequent metastasis. Here we show that plasma redox imbalance caused by albumin oxidation promotes inflammation-independent NETosis. Albumin is the major source of free thiol that maintains redox balance. Oxidation of albumin-derived free thiol is sufficient to trigger NETosis via accumulation of reactive oxygen species within neutrophils. The resultant NETs are found predominantly within lungs where they contribute to the colonization of circulating tumor cells leading to pulmonary metastases. These effects are abrogated by pharmacologic inhibition of NET formation. Moreover, albumin oxidation is associated with pulmonary metastasis in a cohort of head and neck cancer patients. These results implicate plasma redox balance as an endogenous and physiologic regulator of NETosis and pulmonary cancer metastasis, providing new therapeutic and diagnostic opportunities for combatting cancer progression.

## Abstract (web summary)

Neutrophil extracellular traps (NETs) are known to promote metastasis in mouse models. Here the authors show plasma redox imbalance caused by albumin oxidation to induce inflammation-independent NETosis and lung metastasis, and albumin oxidation and reduced plasma free thiol to be associated with lung metastasis in a cohort of head and neck cancer patients.

## Introduction

Neutrophils release web-like DNA-containing structures (i.e., neutrophil extracellular traps [NETs], generated by a process termed NETosis) to entrap pathogens and prevent their dissemination into the circulation^1^. There is emerging evidence from preclinical cancer models that this entrapment effect of NETs is a pathological mechanism for metastasis initiation by promoting colonization of circulating tumor cells (CTCs)^2,3^. These models involve massive systemic inflammation, for example by injection of lipopolysaccharide (LPS)^4^, clamping of hepatic artery and portal vein^3,5^, or cecum ligation^2^. As these perturbations do not faithfully reproduce the clinical state of cancer patients and are non-specific in their mechanisms of inducing NETosis, it remains unknown whether NETs have a major influence on distant metastasis in human cancer.

Accumulation of reactive oxygen species (ROS) within neutrophils is a key process for the initiation of NETosis^6^. Inhibiting this process by antioxidants such as free thiol-containing agents prevents NETosis^7–9^. Amongst the endogenous antioxidants, albumin comprises the largest plasma thiol pool^10^ and thus determines the plasma redox status. The interaction of the free thiol group in albumin with ROS produces oxidized albumin in exchange for catabolizing ROS^11^. Accumulation of oxidized albumin is associated with the progression in various chronic diseases^12–14^, but there is no known role for albumin oxidation in the pathophysiology of cancer.

In this study, we identify endogenous and physiologic regulators of NETosis that act in the absence of massive systemic inflammation, and we define their roles in promoting cancer metastasis. Specifically, we find that plasma redox imbalance caused by albumin oxidation promotes inflammation-independent NETosis and cancer metastasis within the lungs.

## Results

### Albumin oxidation triggers NETs without inflammatory stimuli

**Fig. 1:**
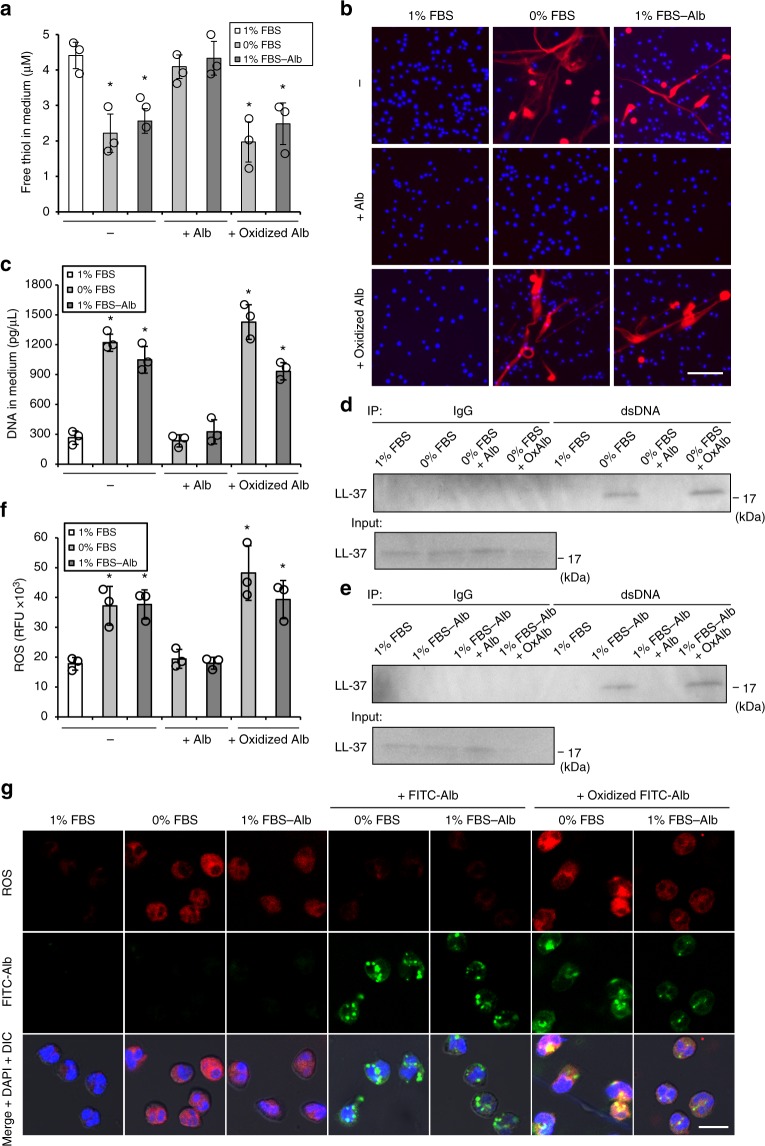
Albumin oxidation triggers NETosis without inflammatory stimuli. **a** Free thiol concentration in medium containing 1% FBS, 0% FBS, and 1% albumin-depleted FBS (1% FBS-Alb) with or without albumin replenishment (final concentration: 0.02 g/dL, matching the albumin concentration in the condition of 1% FBS). **b**–**g** Human neutrophils were cultured under the conditions of 1% FBS, 0% FBS, and 1% FBS-Alb with or without albumin replenishment for 6 h (**b**–**e**), 5 min (**f**), or 1 h (**g**). **b** Representative images of neutrophils stained with cell-permeable DNA dye, Hoechst 33342 (blue), and cell-impermeable DNA dye, SytoxOrange (red). Bar = 50 μm. **c** Concentration of extracellular DNA within culture medium. **d**, **e** Detection of LL-37 physically associated with extracellular DNA within culture medium. Extracellular DNA was immunoprecipitated from culture medium using an anti-dsDNA antibody or IgG isotype control, and the isolated DNA (or the input culture medium without immunoprecipitation) was subjected to Western blotting for LL-37. **f** Intracellular ROS levels within human neutrophils in the indicated culture conditions. **g** Representative images of intracellular ROS and albumin in neutrophils. Intracellular ROS was stained by CellROX Deep Red Reagent. Albumin internalization by neutrophils was visualized using albumin-fluorescein isothiocyanate conjugate (FITC-Alb). Bar = 10 μm. **a**, **c**, **f** Results represent individual values with the mean ± s.d. (*n* = 3; biological replicates, significant differences were compared with control at *P < 0.05 by Dunnett’s test)

In order to investigate a possible role of albumin in regulating NETosis in vitro, we examined the effects of albumin depletion on cultured neutrophils. Human neutrophils were cultured under albumin-containing and albumin-free conditions using medium supplemented with 1% fetal bovine serum (FBS; 1% FBS), no FBS (0% FBS), or 1% albumin-depleted FBS (1% FBS-Alb). The 0% FBS and 1% FBS-Alb conditions displayed approximately half of the free thiol levels compared to 1% FBS (Fig. 1a). Six-hour incubation under both albumin-free conditions led neutrophils to form NETs, characterized by elongated DNA fibers (Fig. 1b) in association with LL-37 (Supplementary Figure 1), which is identical to NETs triggered by inflammation-related stimuli with phorbol 12-myristate 13-acetate (PMA)^15^. Consistent with this observation, levels of extracellular DNA released into the culture medium were elevated in both albumin-free conditions (Fig. 1c, Supplementary Figure 2), and the extracellular DNA was physically associated with LL-37 (Fig. 1d, e). The inhibitory activity of FBS and albumin on NETs was not due to NET degradation (Extended Data Fig. 2), suggesting that albumin blocked NET production. Further supporting this role of albumin in regulating NETosis, a free thiol-containing agent, *N*-acetylcysteine (NAC)^7,8^, and an inhibitor of peptidyl arginine deiminase (PAD), Cl-amidine^16,17^, both blocked NETosis under albumin-free conditions (Supplementary Figure 3a–e); these results indicate that loss of albumin induces NETosis through the established mechanisms of intracellular ROS accumulation and PAD activation.

Based on its known capabilities for ROS scavenging, we reasoned that the free thiol of albumin might be required for inhibiting NETosis. Indeed, although replenishment of bovine serum albumin (BSA) effectively inhibited NETosis under albumin-free conditions (Fig. 1b–e), BSA oxidized by chloramine-T^18^ (Fig. 1a) was incapable of inhibiting NETosis (Fig. 1b–e). Intracellular ROS level, which was elevated in neutrophils cultured under albumin-free conditions (Fig. 1f, g), was maintained at a comparably low level by BSA but not by oxidized BSA (Fig. 1f, g), although uptake of BSA and oxidized BSA by neutrophils was similarly observed (Fig. 1g). Oxidized BSA also triggered NETosis within whole blood (Supplementary Figure 4), indicating that priming and/or pre-activation of neutrophils during the process of neutrophil isolation from whole blood is unlikely to explain the NETosis shown in Fig. 1b. Taken together, free thiol supplied by albumin maintains redox balance in neutrophils, and its oxidation triggers NETosis without inflammation-related stimuli.

### Plasma redox imbalance triggers NETosis

**Fig. 2:**
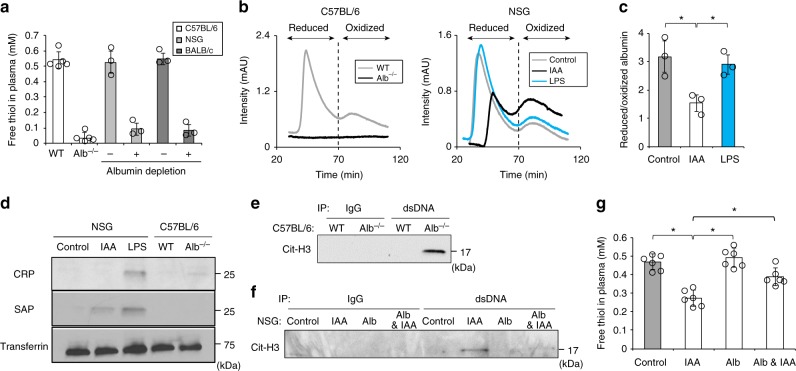
Plasma redox imbalance induces NETosis. **a** Concentration of free thiol in plasma from wild-type (WT) vs. albumin deficient (Alb^−/−^) C57BL/6 mice and control vs. albumin-depleted plasma from NOD scid gamma (NSG) and BALB/c mice. **b**–**d** Plasma isolated from the blood of WT and Alb^−/−^ C57BL/6 mice and NSG mice 2 days post saline, IAA (30 mg/kg), or LPS (8 mg/kg) injection was subjected to FPLC (**b**, **c**) and Western blotting (**d**). **b** Representative FPLC results for the redox state of albumin in C57BL/6 (left) and NSG (right) mice. **c** The ratio of peak intensity for reduced/oxidized albumin in NSG mice. **d** Western blotting with an antibody against C-reactive protein (CRP) and serum amyloid P component (SAP). **e**–**g** Plasma was isolated from the blood of WT and Alb^−/−^ C57BL/6 mice and NSG mice 2 days post saline or IAA (30  mg/kg) injection following intravenous injection of PBS or murine albumin (20 mg/mouse). **e**, **f** Immunoprecipitation of cell-free DNA in the plasma from C57BL/6 (**e**) and NSG (**f**) mice using an anti-dsDNA antibody followed by Western blotting using an antibody against citrullinated histone H3 (Cit-H3). **g** Plasma free thiol concentration. **a**, **c**, **g** Results represent individual values with the mean ± s.d. (*n* = 3–6; biological replicates, *P < 0.05 by Dunnett’s test)

We next examined whether loss of albumin free thiol could be a trigger for NETosis in vivo. First, we assessed the contribution of albumin to total plasma free thiol in mice. Compared with wild-type C57BL/6 mice, mice that were genetically deficient for albumin (Alb^−/−^)^19^ contained approximately 90% less total plasma free thiol (Fig. 2a). Albumin depletion from plasma obtained from other mouse strains (i.e., NSG and BALB/cByJ) confirmed that the vast majority of plasma free thiol was supplied by albumin (Fig. 2a). These results indicate that free thiol provided by circulating albumin determines plasma redox balance in mice.

Next, we tested the association between loss of albumin free thiol and NETosis. For this purpose, we used two different mouse models, Alb^−/−^ mice and Alb^wt/wt^ mice with pharmacological depletion of albumin free thiol by iodoacetamide (IAA)^20,21^. In vitro, IAA-treated BSA was incapable of scavenging ROS within neutrophils and inhibiting resultant NETosis (Supplementary Figure 5a–d), in keeping with the effects of albumin oxidation (Fig. 1c, d). In vivo, IAA administration reduced plasma free thiol levels with the lowest level observed at day 2 following injection (Supplementary Figure 6a) without significantly impacting plasma albumin concentration (Supplementary Figure 6b). The concentration of reduced glutathione in plasma, which only represented approximately 2% of total plasma free thiol, was not significantly altered by IAA administration (Supplementary Table 1), suggesting instead that the effect of IAA administration on plasma free thiol may be due to albumin oxidation. To obtain direct evidence of albumin oxidation by IAA, we performed FPLC to analyze the redox state of albumin. This assay shows albumin-specific signal (Fig. 2b, left) and provides its redox profile (Supplementary Figure 7a–c). Compared to control mice, plasma obtained at day 2 from IAA-injected mice contained less reduced and more oxidized albumin (Fig. 2b, right; Fig. 2c). This oxidative shift of albumin was not observed in the plasma from mice in which systemic inflammation was induced by LPS injection (Fig. 2b, right; Fig. 2c). As expected, LPS injection in these mice led to elevated plasma levels of C-reactive protein (CRP; a commonly used marker of inflammation) and serum amyloid P component (SAP; an acute-phase inflammatory protein in the mouse^22^); in contrast, genetic knockout of albumin and pharmacological depletion of albumin free thiol did not elevate plasma levels of CRP and SAP as much as the induction of systemic inflammation by LPS injection (Fig. 2d). We further identified evidence of NETosis in the plasma from Alb^−/−^ mice and from IAA-injected mice by detecting a NET marker, citrullinated histone H3 (Cit-H3)^23^ in physical association with circulating cell-free DNA (Fig. 2e, f, Supplementary Figure 8a, b). When murine albumin was injected prior to IAA injection, IAA-evoked NETosis was suppressed along with normalization of plasma free thiol level (Fig. 2f, g), indicating that IAA triggers NETosis through albumin oxidation and consequent decrease in plasma free thiol. As an alternative anti-oxidant, NAC was injected prior to IAA administration, but it did not normalize plasma free thiol level (Supplementary Figure 9) possibly due to its low bioavailability^24^. Collectively, plasma redox imbalance caused by lack of albumin free thiol triggers NETosis in the absence of a massive systemic inflammatory response.

### Plasma redox imbalance triggers lung-predominant NETosis

**Fig. 3:**
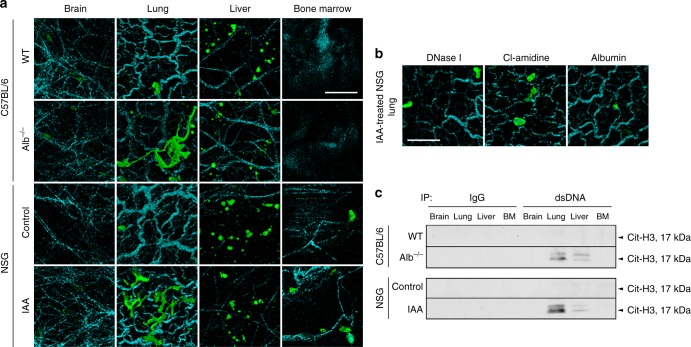
NET-like structures triggered by plasma redox imbalance are lung-predominant. **a**–**c** The indicated organs were resected from WT and Alb^-/-^ C57BL/6 mice and NSG mice 2 days post saline or IAA (30 mg/kg) injection with or without single injection of DNase I on day 2, daily injection of Cl-amidine, or single injection of murine albumin on day 0. **a**, **b** The resected organs were subjected to two-photon microscopy. Extracellular DNA (green) and surrounding collagen-rich parenchyma (cyan) were visualized by SytoxGreen injected via tail vein 20 min before organ harvest and by second-harmonic generation, respectively. **a** Representative images are shown. Bar = 40 μm. **b** Representative images of the lungs from IAA-injected NSG mice with DNase I, Cl-amidine, or albumin treatment. Bar = 40 μm. **c** Extracts of the organs were subjected to immunoprecipitation using an anti-dsDNA antibody followed by Western blotting using an antibody against Cit-H3. Arrows indicate the molecular weight corresponding to Cit-H3

Next, we assessed the distribution of NETs deposited in the organs of mice with plasma redox imbalance. As the brain, lung, liver, and bone are known organs prone to metastasis formation for many cancer types, we assessed these four organs for evidence of NETs deposition in our mouse models of albumin deficiency and oxidation (i.e., Alb^−/−^ mice and IAA-injected Alb^wt/wt^ mice). Two-photon microscopy of these organs demonstrated that NET-like DNA structures stained by the cell-impermeable DNA dye, SytoxGreen, were detected exclusively in the lungs from Alb^−/−^ mice and from Alb^wt/wt^ mice 2 days post IAA injection (Fig. 3a, Supplementary Figure 10a). These NET-like structures were not observed in the lungs of mice treated with DNase I, Cl-amidine, or albumin (Fig. 3b, Supplementary Figure 10b), indicating that these SytoxGreen-positive web-like structures were compatible with NETs. We did not observe NETs within the other organs, including the liver, which instead only displayed round-shaped structures with green autofluorescence (Fig. 3a, Supplementary Figure 10a) even in the absence of SytoxGreen (Supplementary Figure 10c), compatible with vitamin A deposits^25^. In the extracts of the organs, DNA-associated Cit-H3 was enriched in those of the lung from Alb^−/−^ mice and IAA-injected Alb^wt/wt^ mice (Fig. 3c, Supplementary Figure 8c). Collectively, plasma redox imbalance induced by loss of albumin free thiol causes lung-predominant NETosis.

### Plasma redox imbalance leads to lung metastasis via NETs

**Fig. 4:**
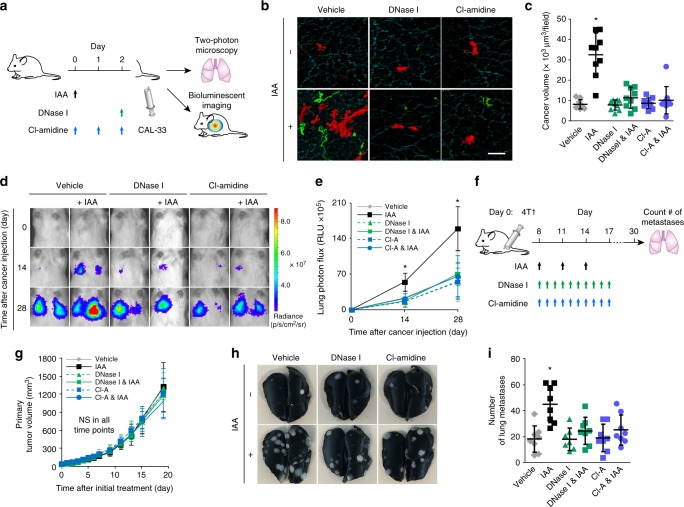
Plasma redox imbalance promotes lung metastasis via NETs. **a** Experimental schema for (**b**–**e**): the effect of NETs induced by albumin-thiol blocking on pulmonary metastasis following tail vein injection of CAL-33 HNSCC cells. Saline or IAA (30 mg/kg) was injected intraperitoneally into NSG mice. DNase I or Cl-amidine was administered at the indicated time points. Two day later, SytoxGreen and CAL-33-mCherry cells (**b**, **c**) or CAL-33-luciferase (**d**, **e**) were injected. **b**, **c** Extracellular DNA (green), CAL-33-mCherry cells (red), and surrounding collagen-rich parenchyma (cyan) within resected lungs were visualized by two-photon microscopy. (**b**) Representative images are shown. Bar = 50 μm. **c** Quantification of the mCherry-positive volume from two-photon microscopy images of resected lungs. **d**, **e** The growth of pulmonary metastases was monitored by bioluminescence imaging. **d** Representative images are shown. **e** Luciferase bioluminescence intensity from the lungs over time (0–28 days). **f** Experimental schema for (**g**–**i**): the effect of NETs induced by albumin-thiol blocking on a spontaneous pulmonary metastasis model. 4T1 murine mammary carcinoma cells were orthotopically transplanted to the mammary fat pads of female Balb/c mice. Saline or IAA (30 mg/kg) was injected intraperitoneally at the indicated time points. Between 8 to 17 days after transplantation, saline, DNase I, or Cl-amidine was administered daily. **g** Primary tumor growth. NS not significant. **h**, **i** Lungs were removed 30 days after transplantation. **h** Representative images are shown. **i** Number of metastatic lung colonies. (**c, i**) Results represent individual values with the mean ± s.d. **e**, **g** Results represent the mean ± s.d. **c** *n* = 9 per group: three fields in three independent experiments. **e** *n* = 5 per group; one of the mice in the DNase I & IAA group died on day 1 for unknown reasons. **g**, **i** *n* = 8 per group; one of the mice in the DNase I group died on day 14 for unknown reasons. *P < 0.05 by Dunnett’s test

We next examined whether lung-predominant NETs generated by plasma redox imbalance promote pulmonary cancer metastasis through NET-dependent entrapping of CTCs. For this purpose, we used the mouse model of albumin oxidation by IAA rather than the Alb^−/−^ mouse because albumin has a wide variety of physiological functions (e.g., maintenance of intravascular pressure, drug transport, etc.)^26^ and its deficiency could affect cancer progression in various aspects. CAL-33 head and neck squamous cell carcinoma (HNSCC) cells with stable expression of either mCherry or *firefly* luciferase (CAL-33-mCherry and CAL-33-Luc, respectively) were intravenously injected into the tail vein of mice with or without IAA administration (Fig. 4a). Two-photon microscopy of ex vivo lungs harvested just after the injection of CAL-33-mCherry demonstrated greater numbers of trapped CTCs in IAA-injected vs. mock-injected mice (Fig. 4b, c). Clusters of CAL-33-mCherry cells were observed in close proximity to SytoxGreen-stained NETs in IAA-injected mice (Fig. 4b), but not in mice treated with DNase I or Cl-amidine to inhibit NET formation (Fig. 4b, c). Longitudinal observation of the trapped CTCs by optical imaging after the intravenous injection of CAL-33-Luc showed that mice with IAA-induced NETs displayed consistently elevated bioluminescent intensity in the lungs, while both DNase I and Cl-amidine reversed the effect of IAA (Fig. 4d, e). Of note, this mouse model of lung metastasis also showed sacrum bone metastasis (Supplementary Figure 11a), but unlike the lung metastases NET induction did not have an effect on bone metastases (Supplementary Figure 11b, c). Administration of IAA did not inhibit the tumor growth of CAL-33 when implanted orthotopically into the tongue of NSG mice (Supplementary Figure 12). Taken together, these results indicate that lung-predominant deposition of NETs following IAA administration specifically promotes pulmonary metastases.

To further confirm these findings, a murine model of spontaneous pulmonary metastasis was utilized, wherein murine 4T1 breast cancer cells were transplanted orthotopically into the mammary fat pad of immunocompetent BALB/cByJ mice (Fig. 4f). Although the administration of IAA did not promote the growth of the primary tumor in the mammary fat pad (Fig. 4g), the number of metastatic colonies was markedly increased by IAA administration (Fig. 4h, i). This effect was rescued by treatment with DNase I or Cl-amidine (Fig. 4h, i), indicating its dependence on NETs. Thus, our results from two distinct pre-clinical models indicate that albumin oxidation-triggered NETs in the lungs promote colonization of CTCs and initiation of pulmonary metastasis.

### Plasma redox imbalance, NETs, and metastasis in human cancer

**Fig. 5:**
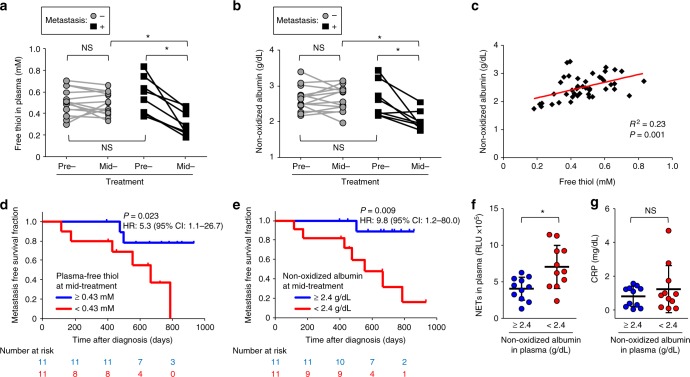
Plasma redox imbalance, NETs, and metastasis in human cancer. **a**–**g** Pre-treatment and mid-treatment plasma samples from HNSCC patients who developed lung metastasis (Metastasis + ; *n* = 8) and matched control patients without metastasis (Metastasis –; *n* = 14) were analyzed for free thiol (**a**), and non-oxidized albumin (**b**) concentration. **c** Relationship between the concentration of plasma free thiol and non-oxidized albumin. The correlation between plasma free thiol and non-oxidized albumin was analyzed (*n* = 44, Pearson’s correlation coefficient test, *R*^2^ = 0.23, *p* = 0.001). **d**, **e** Kaplan–Meier analysis of lung metastasis-free survival in the entire cohort stratified by the median values of plasma free thiol (**d**), and non-oxidized albumin (**e**) concentration at mid-treatment (hazard ratio = 5.3 [95% CI 1.1–26.7] and 9.8 [95% CI 1.2–80.0], respectively; log-rank *p* = 0.023 and 0.009, respectively) (**f**, **g**) Plasma level of NETs (**f**) and CRP (**g**) in the HNSCC patient cohort stratified by the median values of non-oxidized albumin. The data are individual values with the mean ± s.d. (*n* = 11 in each group). *P < 0.05 by (**a**, **b**) repeated-measures ANOVA for split-plot designs and (**f**, **g**) Student’s *t*-test, NS not significant

To investigate the association among plasma redox balance, NETosis, and lung metastasis in human cancer, we analyzed pre-treatment and mid-treatment plasma samples from a cohort of 22 non-metastatic HNSCC patients undergoing curative therapy (Supplementary Table 2). Patients who developed subsequent lung metastasis (*n* = 8; median time-to-metastasis = 489 days [range: 115–786 days]) had a significantly lower concentration of plasma free thiol at mid-treatment compared to the 14 group-matched controls who did not develop metastasis (Fig. 5a; *p* *=* 0.001). Consistent with this result, significantly lower concentration of non-oxidized albumin (i.e., albumin containing a free thiol residue) in plasma was observed amongst the patients who subsequently developed lung metastasis (Fig. 5b; *p* = 0.011). Across the entire cohort, the concentration of non-oxidized albumin was positively correlated with free thiol concentration (*R*^2^ = 0.23, *p* = 0.001; Fig. 5c). Stratified at the median, HNSCC patients with <0.43 mM of free thiol or <2.4 g per dL of non-oxidized albumin at mid-treatment demonstrated significantly inferior lung metastasis-free survival (hazard ratio = 5.3 [95% CI 1.1–26.7] and 9.8 [95% CI 1.2–80.0], respectively; Fig. 5d, e) compared to patients with higher than the median values. Furthermore, a significant increase in NETs was observed in the patients with <2.4 g per dL vs. ≥2.4 g per dL of non-oxidized albumin at mid-treatment (Fig. 5f; *p* = 0.008) while the concentration of total cell-free DNA between these two groups did not show a significant difference (Supplementary Figure 13). Moreover, between these groups, there was no significant difference in plasma concentration of CRP (Fig. 5g). Thus, albumin oxidation-associated decrease in plasma free thiols (i.e., plasma redox imbalance) during treatment could be an independent risk factor for developing elevated levels of NETosis and subsequent lung metastasis.

## Discussion

Until now, physiologic regulation of NETosis in human cancer has remained elusive. We have demonstrated that plasma redox imbalance caused by albumin oxidation triggers NETosis and cancer metastasis without massive inflammation. These results support a model in which the circulating pool of albumin thiols acts as an endogenous and physiologic regulator of intravascular redox balance and NETosis. Our results also revealed a unique anatomical distribution of NET formation—predominantly in the lungs—that may further clarify the clinical significance of this phenomenon in cancer patients. Based on our findings, we propose that NET deposition specifically within the lungs, by leading to entrapping of CTCs, is a previously unrecognized mechanism for lung tropism of cancer metastasis.

In our model, under normal physiologic conditions albumin provides an abundant source of free thiol within plasma that is available to scavenge ROS within circulating neutrophils. However, when this reservoir of albumin thiols becomes depleted in a cancer state, ROS may accumulate within neutrophils leading to NETosis and seeding of hematogenous metastases. Oxidation of albumin thiols within the plasma of cancer patients may occur through multiple mechanisms. In this study, we used mouse models with either exogenous injection of IAA to oxidize albumin or genetic modification to prevent expression of albumin. Although these are not physiological perturbations, we also observed consistent findings in cancer patients subjected to radiotherapy, which is a known and repeated source of ROS generation^27^. This apparent link between redox imbalance and metastasis in human cancer should be interpreted with caution, however, given that our cohort study only demonstrated a correlative relationship and that causation cannot be established from these data alone. Other known physiologic factors impacting plasma redox balance in human cancer patients could include tobacco exposure^28^ or ROS produced by cancer cells^29^ and/or within the tumor microenvironment^30^. As such, in human cancer patients plasma redox imbalance could alternatively reflect early tumor growth as opposed to having a direct causative role in promoting cancer metastasis. These models and their impact on human cancer progression could potentially be distinguished in the future if there were a viable therapeutic strategy for targeting NETs or plasma redox imbalance in humans.

The lung predominance of NET deposition and NET-dependent metastasis were unexpected conclusions of our study. There are likely to be multiple factors that contribute to the lung specificity of this phenomenon. The pulmonary capillary bed is the largest vascular bed in the body and is the predominant site of physiological neutrophil margination^31^. Interleukin-8, a known neutrophil chemoattractant, has been suggested to promote NETosis^32^, is expressed at high levels in healthy lung (GTEx Project)^33^ and diseased lung tissues^34^, and can be induced by ROS^35^, which may create an environment that favors NETosis when additional triggers are present.

Detection of NETs in vivo presents numerous technical challenges. To identify NETs within mouse lungs, we relied on detection of DNA-associated Cit-H3 in tissue extracts and visualization of extracellular DNA with SytoxGreen by 2-photon microscopy. Although SytoxDye-positive NET-like structures have been used previously as a surrogate for NETs in vivo^2,4^, we note that it is a membrane impermeable double-stranded DNA intercalating dye and is not specific for NETs. Supporting our conclusion that these SytoxGreen-positive NET-like structures do indeed represent NETs, they were not visualized when NET production was inhibited by a PAD inhibitor. Future methodologic advances could allow for more specific in vivo visualization of NETs so that they could be distinguished from other extracellular DNA-containing structures.

Our findings have significant implications for future clinical investigations. For instance, additional studies are needed to carefully characterize the relationship between distinct types and stages of human cancer, plasma free thiol levels, albumin redox status, NET markers, environmental and treatment exposures, and sites of cancer metastasis. NET-mediated metastasis likely represents one of a number of mechanisms by which pulmonary metastasis can be initiated, and thus the therapeutic impact of targeting plasma redox balance and/or NETs is unlikely to result in complete reversal or prevention of lung metastasis in all clinical settings. Future work should focus on identifying predictive biomarkers for selecting patients susceptible to NET-mediated pulmonary metastasis.

The relationship between neutrophils and cancer progression has been extensively studied; neutrophils may participate in promotion of cancer metastasis through multiple mechanisms^36^, but therapeutic targets that modulate neutrophil activity have not been established. Park et al.^37^ proposed that DNase I could inhibit lung metastasis in a mouse model of breast cancer. Cedervall et al.^38^ suggested that DNase I and PAD inhibitors could counteract NET-mediated thrombosis and organ failure as well as cancer metastasis. Herein, we propose that cancer patients at elevated risk of lung metastasis might benefit from anti-NET therapies, such as DNase I or PAD inhibitors. This strategy has the potential for broad applications in oncology since, as our results demonstrate, even cancer patients without massive inflammation may still be prone to develop NETs and NET-mediated lung metastasis. Levels of plasma free thiol and/or non-oxidized albumin may provide a potentially generalizable biomarker that can assess the efficacy of such drugs in reversing the detrimental effects of dysregulated redox balance. Moreover, plasma redox balance may represent a novel and promising therapeutic target for NET inhibition and metastasis prevention. Together, the anti-NET therapies and potential predictive biomarkers described here could provide a potent clinical strategy to combat cancer metastasis.

## Methods

### Neutrophil isolation

Human whole blood from healthy volunteers was collected into ethylenediaminetetraacetic acid (EDTA)-containing tube. Neutrophils were isolated by density gradient centrifugation using Polymorphprep (Alere Technologies, Germany) according to the manufacturer’s instructions. Neutrophils were re-suspended with Roswell Park Memorial Institute (RPMI) 1640 (Gibco/Life Technologies, MA, USA) without phenol red supplemented with 1% fetal bovine serum (FBS; Gibco/Life Technologies, MA, USA) for normal culture conditions. Alternatively, neutrophils were cultured under serum-free (i.e., RPMI-1640 with 0% FBS) or albumin-depleted (i.e., RPMI-1640 with 1% FBS without albumin—see “Albumin depletion from serum or plasma”) conditions. For the replenishment of albumin, bovine serum albumin (BSA; BioShop Canada Inc., Canada) dissolved in phosphate buffered saline (PBS) was freshly prepared and added to albumin-free media at a final concentration of 0.02 g per dL, matching the albumin concentration in the condition of 1% FBS. Neutrophil purity was established to be routinely >90%, as assessed by May-Grünwald Giemsa (Sigma-Aldrich, Canada) staining.

### Cell culture

The human head and neck squamous cell carcinoma (HNSCC) cell line CAL-33 was a kind gift from Dr. Jennifer Grandis (University of Pittsburgh). The murine breast cancer cell line 4T1 and the embryonic human kidney cell line HEK293T were kind gifts from Dr. Graham Fletcher (Campbell Family Institute for Breast Cancer Research, Canada) and Dr. Bradly Wouters (Princess Margaret Cancer Centre, Canada), respectively. CAL-33 and HEK293T were cultured in 10% FBS-containing Dulbecco’s modified Eagle’s medium (Gibco/Life Technologies, MA, USA), and 4T1 cells were cultured in 10% FBS-containing RPMI-1640 in a well-humidified incubator with 5% CO_2_ and 95% air at 37 °C.

### Albumin depletion from serum and plasma

Albumin was depleted from serum or plasma using polystyrene columns (ThermoFisher Scientific, MA, USA) packed with albumin-binding resin (CaptureSelect MultiSpecies Albumin Depletion; ThermoFisher Scientific, MA, USA). Five hundred microliters of serum or plasma was loaded into the column packed with 2.5 mL of resin and washed with PBS by gravity flow. These processes were repeated 2–4 times until the concentration of albumin yields less than 0.2 g per dL. Flow-through fractions were collected and immediately passed through a 0.22 μm sterile filter (MilliporeSigma, MA, USA) to remove bacterial contamination. The degree of albumin depletion was confirmed by quantifying the concentration of total protein and albumin with the Bradford protein assay (Bradford Reagent, Sigma-Aldrich, Canada) and bromocresol green assay (BCG albumin assay kit, Sigma-Aldrich, Canada), respectively. Adsorbed albumin was eluted with 0.1 M glycine, pH 3.0.

### Induction and inhibition of NETosis in vitro

For the induction of NETosis, human neutrophils (5 × 10^5^) cultured with RPMI-1640 containing 0% FBS, 1%FBS without albumin, or 1% FBS with 100 nM of phorbol 12-myristate 13-acetate (PMA; Cayman Chemical, MI, USA). For the inhibition of NETosis, human neutrophils (5 × 10^5^) were cultured with albumin-free media containing 0.02 g per dL of BSA, 1 mM of *N*-acetylcysteine (NAC; Sigma-Aldrich, Canada), or 50 μM of Cl-amidine (MilliporeSigma, MA, USA). For NET inhibition by Cl-amidine, neutrophils in the media containing 1% FBS were pre-treated with 50 μM of Cl-amidine for 30 min. After six-hour incubation, the culture medium was collected after vigorous agitation. The medium was centrifuged at 100 × *g* for 1 min and the supernatant was used for the quantification of NETs.

### Quantification of extracellular and cell-free DNA

The concentrations of total extracellular DNA in culture medium and circulating cell-free DNA in plasma were determined by quantitative polymerase chain reaction (qPCR) with primers targeting the second open reading frame of the long interspersed element-1 transposon (LINE-1) (forward/reverse primer for human and mouse LINE-1: 5′-TCACTCAAAGCCGCTCAACTAC-3′/5′-TCTGCCTTCATTTCGTTATGTACC-3′ and 5′-AATGGAAAGCCAACATTCACGTG-3′/5′-CCTTCCTTGACCAAGGTATCATTG-3′, respectively; Eurofins Genomics, Belgium). The culture medium and plasma were diluted 1:100 with DNA suspension buffer (TEKnova, CA, USA). The reaction mixture for each qPCR consisted of 4 μL of a diluted sample, 1 μL of 2.5 μM LINE-1 primer mixture, and 5 μL of Sso Advanced Universal SYBR Green Supermix (Bio-Rad Laboratories, CA, USA). Real-time PCR amplification was performed with a pre-cycling heat activation of DNA polymerase at 98 °C for 3 min followed by 40 cycles of denaturation at 98 °C for 10 s, and annealing and extension at 60 °C for 30 s using a CFX384 Touch Real-Time PCR Detection System (Bio-Rad Laboratories, CA, USA). Absolute quantification of DNA in each sample was determined by a standard curve with serial dilutions of human genomic DNA (Promega, WI, USA).

### Imaging of NETs in vitro

Human neutrophils cultured under the designated condition for 6 h were fixed with 4% paraformaldehyde (PFA) for 10 min and permeabilized with PBS containing 0.2% Tween20 for 10 min. After washing with PBS three times and incubating for 20 min in the SuperBlock (PBS) Blocking Buffer (ThermoFisher Scientific, MA, USA), cells were treated with a rabbit anti-LL-37 antibody (1:1000; OSC00009W, Osenses Pty Ltd., Australia) for 1 h at room temperature. After washing with PBS containing 0.1% Tween20 three times, LL-37 was labeled with Alexa Fluor 488 anti-rabbit IgG secondary antibody (1:1000; Invitrogen Inc., CA, USA) for 1 h at room temperature. After an additional three washes in PBS containing 0.1% Tween20, DNA was stained with DAPI (NucBlue Fixed Cell ReadyProbes Reagent, ThermoFisher Scientific, MA, USA). Slides were mounted in ProLong Gold antifade reagent (ThermoFisher Scientific, MA, USA). Images were acquired with a Zeiss LSM700 confocal microscope (Carl Zeiss, Germany). Separately, for staining of live/dead neutrophils and NETs, Hoechst33342 (2 drops per mL; NucBlue Live Cell ReadyProbes Reagent, ThermoFisher Scientific, MA, USA) and SytoxOrange (1:1000; Invitrogen Inc., CA, USA) were added into the culture medium 6 h after initiation of treatment. Neutrophils were then observed with EVOS FL Cell Imaging System (Life technologies, CA, USA).

### Immunoprecipitation and Western blotting

Mouse monoclonal antibodies against double-stranded DNA (ab27156, Abcam, MA, USA) and IgG (12-371, MilliporeSigma, MA, USA) were conjugated to magnetic beads following the manufacturer’s protocol (Dynabeads Antibody Coupling Kit, ThermoFisher Scientific, MA, USA). The mixture of 100 μL of culture medium or mouse plasma and 25 μL of antibody-conjugated beads solution or 500 μL of extracts derived from 20 mg of mouse organs (i.e., brain, lung, liver, and bone marrow from femur and 50 μL of antibody-conjugated beads solution were incubated with shaking for 1 h at room temperature. The supernatant was removed by a magnet. The residual pellet was washed with PBS and re-suspended with 25 μL of 2x Laemmli Sample Buffer (Bio-Rad Laboratories, CA, USA) and boiled for 5 min at 95 °C. Sample solution was separated from the magnetic beads and 20 μL was loaded onto a 4–20% SDS-PAGE gel (Bio-Rad Laboratories, CA, USA), transferred to PVDF membranes (Bio-Rad Laboratories, CA, USA), then blocked in 2.5% skim milk for 1 h. After washing three times with Tris-buffered saline containing 0.1% Tween 20 (TBST) for 5 min each, the membranes were incubated with rabbit polyclonal anti-LL-37 antibody (1:1000, OSC00009W, Osenses Pty Ltd., Australia) for 1 h at room temperature, or rabbit polyclonal anti-histone H3 (citrulline R2 + R8 + R17) antibody (1:500, ab5103, Abcam, MA, USA) overnight at 4 °C. After washing three times with TBST for 5 min each, the membranes were incubated with horseradish peroxidase conjugated anti-rabbit IgG (1:2000; 7074S, Cell Signaling Technology, MA, USA) for 1 h at room temperature. Membranes were washed three times with TBST, developed with Clarity Western ECL Substrate (Bio-Rad Laboratories, CA, USA) and exposed to X-ray film (HyBlot CL Autoradiography Film, Denville Scientific Inc., MA, USA). For the loading controls and input controls, 14 μL of culture medium and 3.5 μL of 4x Laemmli Sample Buffer, 0.4 μL of plasma diluted with 14.6 μL of PBS and 5 μL of 4x Laemmli Sample Buffer, or 2.5 μL of organ extract diluted with 12.5 μL of PBS and 5 μL of 4x Laemmli Sample Buffer was loaded into each well, and then these gels were subjected to immunoblotting as described above. We used LL-37 (1:1,000, OSC00009W, Osenses Pty Ltd., Australia), histone H3 (citrulline R2 + R8 + R17, 1:500, ab5103, Abcam, MA, USA), transferrin (1:500, ab82411, Abcam, MA, USA), β-actin (1:200, sc-47778 HRP, Santa Cruz, CA, USA) where indicated. Full size images of blots are shown in Supplementary Figures 14–19.

### Intracellular ROS detection

Intracellular ROS were measured by the DCFDA Cellular ROS detection Assay Kit (Abcam, MA, USA) according to the manufacturer’s protocol. Isolated human neutrophils were treated with 20 μM DCFDA for 30 min. After centrifugation (400 × *g* for 5 min), neutrophils were re-suspended with the designated medium (i.e., RPMI-1640 with 1% FBS, 0% FBS, and 1% albumin-depleted FBS with or without 0.02 g per dL of BSA/oxidized BSA or 1 mM of NAC) and seeded onto 96-well plates at 1 × 10^5^ cells per well. The ROS level represented by the fluorescence intensity of DCFDA was quantified using a fluorescence plate reader (TECAN Infinite M200Pro, Tecan Group Ltd., Switzerland).

### Imaging of intracellular ROS and albumin in neutrophils

Human neutrophils (5 × 10^5^) were cultured on glass coverslips in 24-well culture plates under the designated condition for 30 min. For visualization of the internalization of supplemented BSA by neutrophils, we added albumin-fluorescein isothiocyanate conjugate (Sigma-Aldrich, Canada) in 1:9 ratio to unlabeled BSA. This BSA mixture was added into the culture medium at final concentration of 0.02 g per dL. To stain intracellular ROS, CellROX Deep Red Reagent (5 μM; ThermoFisher Scientific, MA, USA) was added into the culture medium. After washing three times with PBS, neutrophils were fixed with 4% PFA for 20 min. After an additional washing three times with PBS, DNA was stained with DAPI (NucBlue Fixed Cell ReadyProbes Reagent, ThermoFisher Scientific, MA, USA). Slides were mounted in ProLong Gold antifade reagent (ThermoFisher Scientific, MA, USA). Images were acquired with a Zeiss LSM700 confocal microscope (Carl Zeiss, Germany).

### Lentiviral transfection

The packaging plasmid (psPAX2), the envelope plasmid (PMD2G), and pLenti-CMV-Puro-LUC were kind gifts from Dr. Bradly Wouters (Princess Margaret Cancer Centre). pCDH-CMV-mCherry-T2A-Puro was a kind gift from Kazuhiro Oka (plasmid # 72264; Addgene, MA, USA). For lentiviral production, HEK293T cells were co-transfected with 21 μg of psPAX2, 10.5 μg of PMD2G, and 21 μg of pCDH-CMV-mCherry-T2A-Puro or pLenti-CMV-Puro-LUC using the Lipofection transfection reagent (Invitrogen Inc., CA, USA). High-titer viral solutions were prepared and used for transduction into CAL-33 cells. Antibiotics selection with puromycin (2.0 μg per mL, Gibco) was performed for 5 days.

### Blocking of free thiol in albumin in vitro

In order to oxidize BSA, PBS solution of BSA (0.1 g per dL) was incubated with 100 mM of chloramine-T (Sigma-Aldrich, Canada) or 375 mM of iodoacetamide (IAA; Sigma-Aldrich, Canada) for 1 h at 37 °C under oxygen-saturated conditions. In order to remove excess chloramine-T or IAA from the albumin solution, the mixture was centrifuged with Amicon Ultra 10 K centrifugal filter device (MilliporeSigma, MA, USA) and re-suspended with 450 μL of PBS, repeated three times.

### Detection of free thiol in albumin solution and plasma

Culture medium (×1) or human/mouse plasma (×100 dilution with PBS (pH 8.0)) containing 0.1 mM 5,5′-Dithiobis(2-nitrobenzoic acid) (Sigma-Aldrich, Canada) was incubated at 27 °C for 30 min. Absorbance at 412 nm was measured with a spectrophotometer. For calculation of free thiol concentration, molar extinction coefficient of 14.15 mM^−1^ cm^−1^ at 412 nm was used^39^.

### Quantification of plasma concentration of albumin

The mixture of 2.5 μl of plasma, 2.5 μl of water, and 200 μl of BCG albumin assay reagent (BCG albumin assay kit, Sigma-Aldrich, Canada) was incubated in a 96-well plate for 5 min at room temperature. The absorbance at 620 nm was measured using a plate reader (TECAN Infinite M200Pro, Tecan Group Ltd., Switzerland); absolute quantification was determined by a standard curve with serial dilutions of albumin.

### Albumin-deficient mouse model

Albumin-deficient strain (C57BL/6J-Alb^em8Mvw^/MvwJ) was purchased from Jackson Laboratory (ME, USA) as homozygote breeding pairs and maintained at the animal facility in Princess Margaret Cancer Centre. Wild-type control C57BL/6 mice were obtained from the Princess Margaret Cancer Centre Small Animal Facility accredited by the Canadian Council on Animal Care. The plasma or organs from 5–8-week old male and female mice were used.

### Induction of NETosis in vivo

Saline solution of IAA (3 mg per mL, 30 mg per kg of body weight) or LPS (1 mg per mL, 8 mg per kg of body weight; Sigma-Aldrich, Canada) was injected intraperitoneally into 8-week old NOD scid gamma (NSG) mice, which were bred in-house at the Princess Margaret Cancer Centre Small Animal Facility accredited by the Canadian Council on Animal Care. Mice in the vehicle control group were administered the same volume of saline. Where indicated, murine albumin isolated from NSG mouse plasma (see “Albumin depletion from serum or plasma”) was injected into the tail vein (20 mg of murine albumin in 200 μL of PBS) prior to the saline/IAA injection. Blood was collected from the submandibular plexus (Supplementary Figure 6a, b) or via cardiac puncture (Fig. 2b–g) at designated time points. Immediately after blood collection, plasma was isolated by centrifugation (2500 × *g* for 10 min at 4 °C). To eliminate cellular debris, an additional centrifugation was performed (16,100 × *g* for 10 min at 4 °C); isolated plasma was stored at −80 °C until analysis.

### Quantification of the oxidized/non-oxidized forms of albumin

Ten microliters of human and mouse plasma were diluted with 20 μL of 0.10 M sodium phosphate, 0.3 M NaCl (pH 6.87) and filtered through a Spin-X Centrifuge Tube Filters (VWR, PA, USA). Fast protein liquid chromatography (FPLC) analysis was performed using ÄKTA FPLC (GE Healthcare, UK). Albumin was separated on a Shodex Asahipak ES-502N column (Showa Denko Co., Ltd., Japan) at 35 °C. Elution was performed by means of a linear gradient with increasing ethanol concentrations from 0% to 5% for human albumin and from 0% to 1% for mouse albumin in 0.05 mol per L sodium acetate and 0.40 mol per L sodium sulfate mixture (pH 4.85) at a flow rate of 1.0 mL per min. The obtained FPLC profiles for human albumin were subjected to Gaussian curve fitting by Igor Pro 7 (WaveMetrics, OR, USA). The fractions for oxidized and non-oxidized albumin were estimated by dividing the area of each fraction by the total area. Plasma concentration of each form of albumin was calculated by multiplying the fraction for each form of albumin by the plasma concentration of total albumin. As the fractions for reduced and oxidized murine albumin were not separated enough to be analyzed by Gaussian curve-fit algorithms, we utilized the ratio of peak intensity for reduced/oxidized albumin to assess the redox state of murine albumin.

### Quantification of the oxidized/reduced forms of glutathione

Total and oxidized glutathione was measured by GSH/GSSG-Glo Kit (Promega, WI, USA) following the manufacturer’s protocol with the following modifications. First, plasma was loaded onto an Amicon Ultra 10 K centrifugal filter device (MilliporeSigma, MA, USA, 15,000 × *g* for 30 min at 4 °C) for filtration. Next, a mixture of 2.2 µL filtered plasma and 19.8 µL saline (22 µL total) was loaded onto a 96-well microtiter plate (ThermoFisher Scientific, MA, USA). Twenty-two microliter of total or oxidized glutathione reagent (100 mM of *N*-Ethylmaleimide was included in the oxidized glutathione reagent) supplied by the kit was then added onto each well. After five minutes of shaking the plate, 44 μL of luciferin generation reagent supplied by the kit was added onto each well. After thirty minutes of incubation, luciferin detection reagent supplied by the kit was added onto each well and the plate was equilibrated for 15 min. The intensity of chemiluminescence was measured using a plate reader (TECAN Infinite M200Pro, Tecan Group Ltd., Switzerland). The absolute concentrations of total and oxidized glutathione were calculated with the use of standard curves of glutathione supplied by the kit.

### Lung and sacrum metastasis model

As shown in Fig. 4a, to induce NETosis, IAA (3 mg per mL saline, 30 mg per kg of body weight) was injected intraperitoneally into 6–8-week-old NSG male mice 2 days prior to the tail vein injection of CAL-33-luciferase (1 × 10^6^ cells in 200 μL PBS). For anti-NET therapies, a single intravenous injection of bovine pancreas DNase I (3000 units in 100 μL of saline; Sigma-Aldrich, Canada) was administered on day 2; 6-h prior to injection of cancer cells. Cl-amidine (3 mg per mL PBS, 10 mg per kg body weight; MilliporeSigma, MA, USA) was injected intraperitoneally on days 0, 1, and 2. Mice in the vehicle control group were administered the same volume of saline and PBS for each treatment.

### Orthotopic tongue tumor model

CAL-33-luciferase (5 × 10^4^ cells in 30 μL PBS) was orthotopically injected into the tongue of NSG mice under isoflurane anesthesia. Three days after tumor implantation, saline or IAA was injected intraperitoneally into mice every three days. The growth of tongue tumor was monitored by bioluminescence imaging (0–14 days). Fifteen days after tumor implantation, mice became unable to intake food due to the growth of the tongue tumor and thus were euthanized.

### Microcomputed tomography of mice

A GE eXplore Locus Ultra microCT (GE Healthcare, GB) was used for data-acquisition in prone position under isoflurane inhalation (a 16-s Anatomical Scan Protocol (total of 680 images) at 80 kV, 50 mA, using a 0.15-mm Cu Filter, to achieve approximately 150 µm resolution). STTARR’s GPU Reconstruction UI program (in-house software) was used to reconstruct CT scans in DICOM format. Images were analyzed by Siemens Inveon Research Workplace 4.0 (Siemens, PA, USA).

### Lung metastasis model of orthotopic breast cancer

The suspension of 4T1 cells (1 × 10^5^ in 50 μL PBS) was injected into the right fifth mammary fat pad of 6–8-week-old Balb/c female mice (Jackson Laboratory, ME, USA). Eight days after tumor implantation, when tumor became palpable, mice were grouped by block randomization according their primary tumor size. Intraperitoneal injection of IAA (3 mg per mL saline, 30 mg per kg body weight) or saline was initiated on day 8 and repeated on days 11 and 14 (Fig. 4f). As anti-NET therapies, daily intraperitoneal injection of Cl-amidine (3 mg per mL PBS, 10 mg per kg body weight) or daily intravenous injection of DNase I (1200 units in 100 μL of saline) was performed until day 17. Mice in the vehicle control group were administered the same volume of saline and PBS for each treatment. At the first day of IAA or saline injection, Cl-amidine was administered 6-h prior to injection of IAA or saline. Primary tumor volume was calculated as 0.5 × length × width^2^. Thirty days later, mice were euthanized and lungs were removed. A 20-gauge needle was inserted into the trachea and the lung was gradually inflated using 1 ml of PBS solution with India ink (15% (v/v); Speedball, NC, USA). For de-staining, the lung was immersed in Fekete’s solution (100 ml 70% ethanol (Sigma-Aldrich Canada), 10 ml 4% formaldehyde (Sigma-Aldrich Canada) and 5 ml 100% glacial acetic acid (Fisher Scientific, PA, USA). The metastatic nodules were counted by visual observation.

### Imaging of luminescence in lung and sacrum metastases

Optical in vivo imaging was performed using the Xenogen IVIS Spectrum (Caliper Life Sciences Inc., MA, USA). Bioluminescent images were acquired 9 min after intraperitoneal injection of luciferin (150 mg per kg body weight, XenoLight D-Luciferin - K^+^ Salt Bioluminescent Substrate, PerkinElmer, MA, USA). During imaging, mice were anesthetized with 2% isoflurane gas in the oxygen flow (1.5 L per min). Signal intensities were quantified and analyzed using Living Image Software version 4.5 (PerkinElmer, MA, USA).

### Imaging of NETs in the mouse organs

PBS solution of SytoxGreen (5 μL in 200 μL PBS; Invitrogen Inc., CA, USA) was injected into the tail vein of WT and Alb^−/−^ C57BL/6 mice or NSG mice 2 days post saline, IAA (30 mg per kg) injection, followed by 5 × 10^5^ CAL-33-mCherry cells in PBS 20 min later (Fig. 4b, c). Mice were then euthanized and organs (i.e., brain, lungs, liver, and femur) were immediately resected. Femur was cut in half along the long axis and bone marrow was exposed. Two-photon imaging was conducted using a Zeiss LSM710 (Carl Zeiss, Germany) coupled with a W Plan-Apochromat 20×/1.0 DIC water immersion objective (Carl Zeiss, Germany) and a Chameleon femtosecond laser (Coherent Inc., CA, USA). The excitation wavelength was set to 810 nm. SytoxGreen- and mCherry-emitted photons were collected using two emission filters and a beam splitter. For visualizing collagen structures, signals for second-harmonic generation were recorded between 400–410 nm. To visualize three-dimensional structure of NETs, Z-stack images were obtained at 3.0 μm intervals for 7–10 Z-steps (Figs. 3a, b, 4b, Supplementary Figure 10a–c) or 1.5 μm intervals for 20 Z-steps (Fig. 4c) and processed with the ZEN software (Carl Zeiss, Germany). Tumor-infiltrated volume in the lung was quantified as the mCherry-positive volume by Image J software (NIH, MD, USA).

### Human HNSCC patient plasma analysis

All studies involving HNSCC patient specimens were approved by the Research Ethics Board of University Health Network. Informed consent was obtained from all human participants. Patients with oropharyngeal squamous cell carcinoma or HNSCC of unknown primary treated with definitive intensity-modulated radiotherapy or chemoradiotherapy between 2014 and 2015 were identified from a prospective Anthology of Clinical Outcomes^40^. Patients with previous surgery, chemotherapy, or radiotherapy for HNSCC were excluded. Serial blood samples collected in EDTA tubes were obtained prior to commencement of treatment, and at mid-treatment. Each blood sample was processed within 2 h by centrifugation (2500 × *g* for 10 min at 4 °C) followed by aliquoting of plasma and storage at −80 °C until analysis. Samples were analyzed from eight patients who experienced distant relapse in the lung, and 14 control patients without relapse selected by propensity score matching for age, gender, clinical cancer stage, and treatment modality (i.e., radiotherapy or chemoradiotherapy).

### Quantification of NETs in human plasma

We utilized a previously established ELISA assay for the detection of elastase-DNA complexes^41^ with slight modifications. A rabbit polyclonal anti-neutrophil elastase antibody (1:500; ab131260, Abcam, MA, USA) in 100 mM of bicarbonate/carbonate coating buffer (50 μL in total) was coated onto 96-well microtiter plates (ThermoFisher Scientific, MA, USA) overnight at 4 °C. After blocking in 1% BSA for 1 h, the mixture of 50 μL of human plasma and 50 μL of PBS containing 0.3% BSA, 0.05% Tween 20, and 5 mM EDTA was loaded per well and incubated at room temperature for 1 h. After washing three times with PBS containing 0.05% Tween 20 and 5 mM EDTA (PBST-EDTA), a horseradish peroxidase conjugated anti-DNA antibody (1:100; D5425–3–100, Zymo Research Corporation, CA, USA) in PBST-EDTA (100 μL in total) was added to each well and incubated at room temperature for 1 h. After washing three times with PBST-EDTA, the peroxidase substrate (Glo Substrate Reagent Pack (DY993), Bio-Techne Corporation/R&D Systems, MN, USA) was added. The intensity of chemiluminescence was measured using a plate reader (TECAN Infinite M200Pro, Tecan Group Ltd., Switzerland). For analysis, the luminescence of the blank well was subtracted from the luminescence of each sample.

### Detection of inflammatory proteins in plasma

For detecting serum amyloid P component (SAP) or C-reacting protein (CRP) in mouse plasma, 0.4 or 2 μL of plasma diluted with 14.6 or 16 μL of PBS and 5 or 6 μL of 4x Laemmli Sample Buffer (Bio-Rad Laboratories, CA, USA) was sonicated for 15 min and boiled for 10 min at 95 °C. This mixture was subjected to Western blotting (see “Immunopreciptation and Western blotting”) using antibodies against SAP (1:1,000, AF2558, Bio-Techne Corporation/R&D Systems, MN, USA) and CRP (1:200, ab65842, Abcam, MA, USA). Concentration of CRP in human plasma was measured by Human C Reactive Protein ELISA Kit (Abcam, MA, USA) according to manufacturer’s protocol. Samples were diluted 1:50,000 and measured in duplicates.

### Statistical analyses

Data are presented as mean and standard deviation (s.d.) or individual values with or without mean and s.d.. The significance of difference between two independent subjects and among multiple subjects was determined using the Student’s *t*-test and Dunnett’s test, respectively. Differences over time were repeated-measures ANOVA for split-plot designs. Correlation was analyzed using the Pearson correlation coefficient test. The Kaplan–Meier method was used to evaluate distant-metastasis-free survival, and log-rank tests compared survival proportions among groups. Data were obtained from at least three independent experiments with 1–3 technical replicates. Biological replicates represent samples from different sources, while technical replicates represent an assay of samples from the same source. Western blot was performed twice. For the lung metastasis mouse model shown in Fig. 4g–i, mice were grouped by block randomization according their primary tumor size before the initiation of the treatment. Other experiments were not randomized and there was no blinding. All tests were two-tailed with a *P* value < 0.05 considered to be significant. All calculations and analyses were performed in R (version 2.8.1; [http://www.R-project.org]).

### Ethics of animal experiments

All animal experiments were approved by the Animal Research Committee of Princess Margaret Cancer Centre and performed according to guidelines governing animal care in Canada.

## Electronic supplementary material

Supplementary Information

Reporting Summary

## Data Availability

All relevant data are available from the authors. A Reporting Summary for this article is available as a Supplementary Information file.
