# Supplementary material for: Plasma redox imbalance caused by albumin oxidation promotes lung-predominant NETosis and pulmonary cancer metastasis
Source: Nat Commun. 2018 Nov 30;9:5116. doi: 10.1038/s41467-018-07550-x (PMC6269536; doi:10.1038/s41467-018-07550-x)
Supplement: Supplementary file 1 — Supplementary Information [file 41467_2018_7550_MOESM1_ESM.pdf]

Supplementary information

Plasma redox imbalance caused by albumin oxidation promotes lung-predominant NETosis and pulmonary cancer metastasis

Inoue et al.

Supplementary Table 1. The concentration and redox status of glutathione in plasma with or without iodoacetamide treatment.

	**GSH + GSSG**	**GSSG**	**GSH**	**GSH/GSSG**
**Saline**	70.3 ± 0.5	30.0 ± 0.61	10.3 ± 0.93	0.34 ± 0.04
**IAA**	76.0 ± 1.3*	33.4 ± 0.94*	9.2 ± 0.92	0.28 ± 0.03

Units: micromolar. GSH: reduced glutathione. GSSG: oxidized glutathione. Each value is the mean ± s.d. (*n* = 3 in each group). *P<0.05 by Student's *t*-test.

Supplementary Table 2. Patient characteristics.

Characteristics	No metastasis (n = 14)	Metastasis (n = 8)	*p*-value[^]
**Age, median (range)**	59 (33 - 75)	58 (46 - 71)	1
**Sex**			1
**Male**	11	6	
**Female**	3	2	
**Primary site**			1
**Oropharynx**	13	7	
**Unknown**[†]	1	1	
**Stage**			0.66
**II**	1	0	
**III**	0	1	
**IVA**	12	6	
**IVB**	1	1	
**p16 status**[§]			0.25
**Positive**	12	5	
**Negative**	1	3	
**Treatment**			
**Radiotherapy**	6	3	1
**Chemoradiotherapy**	8	5	

**Months follow-up, median (range)**	22 (15 – 29)	16 (6 - 26)	0.09

^Age, Median follow-up: Wilcoxon rank sum test. Sex, Primary site, Stage, p16 status, and Treatment: Fisher exact test.

†Head and neck squamous cell carcinoma of unknown primary defined as the presence of cancer involving one or more lymph nodes within the head and neck region without an identifiable primary tumor.

§ p16 status for one patient in no metastasis group was not tested and thus unknown.

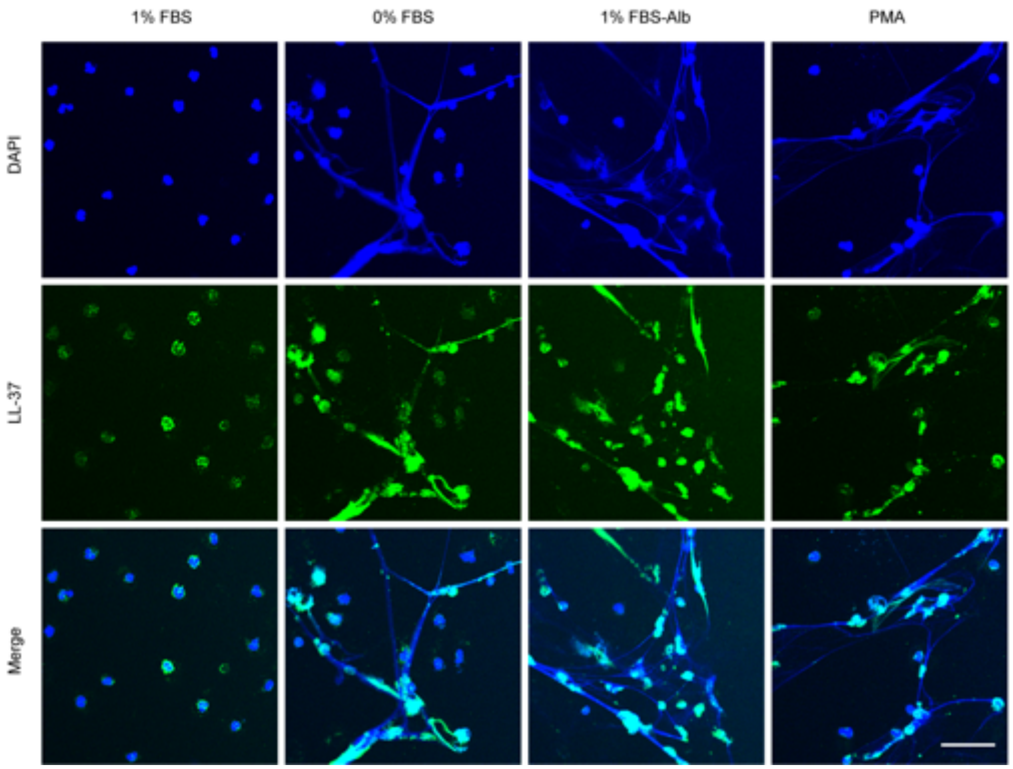

Supplementary Figure 1. Immunocytochemistry for human neutrophils with or without albumin depletion. Human neutrophils were cultured in medium containing 1% FBS, 0% FBS, and 1% albumin-depleted FBS (1% FBS-Alb) for 6 hours. As a positive control for neutrophils undergoing NETosis, human neutrophils cultured in medium containing 1% FBS were treated with 100 nM of phorbol 12-myristate 13-acetate (PMA) for 6 hours. Representative immunofluorescence images are shown of neutrophils stained with DAPI (blue) and anti-LL37 (green). Bar = 50 μm.

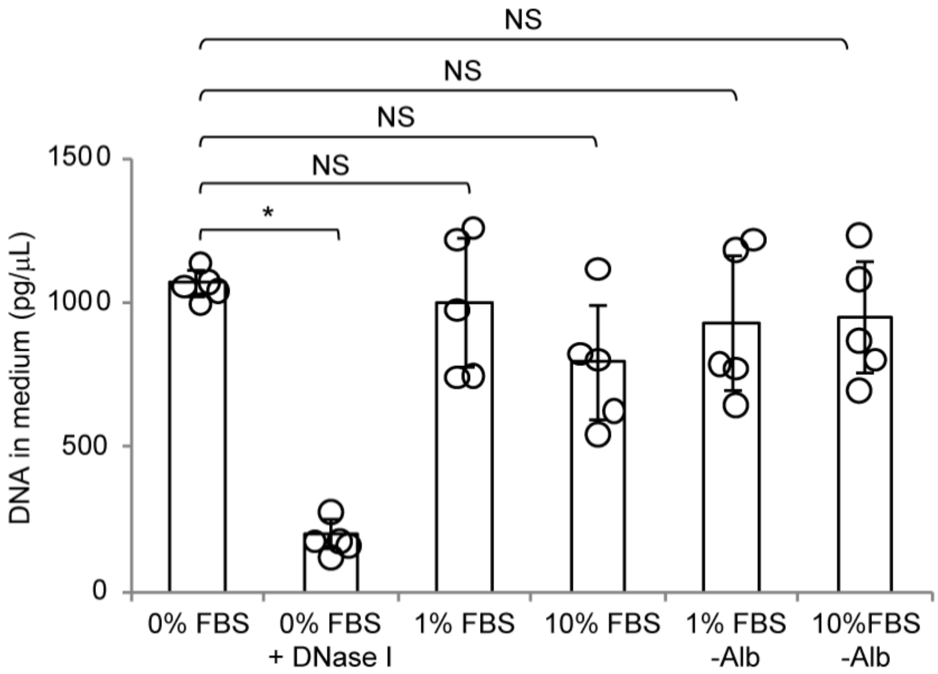

Supplementary Figure 2. The effect of nucleases in FBS on the degradation of NETs. NET-containing medium (obtained from the culture of human neutrophils under 0% FBS condition) was mixed with FBS or albumin-depleted FBS (FBS-Alb) at a final concentration of 1% FBS, 10% FBS, 1% FBS-Alb, and 10% FBS-Alb were incubated at 37°C for 3 hours. Exogenous DNase I (100 units) in media lacking FBS (0% FBS + DNase I) was a positive control for the susceptibility of NETs to degradation by nucleases. Results represent individual values with the mean ± s.d. (*n* = 5; biological replicates. NS = not significant. **P*<0.05 by Dunnett's test).

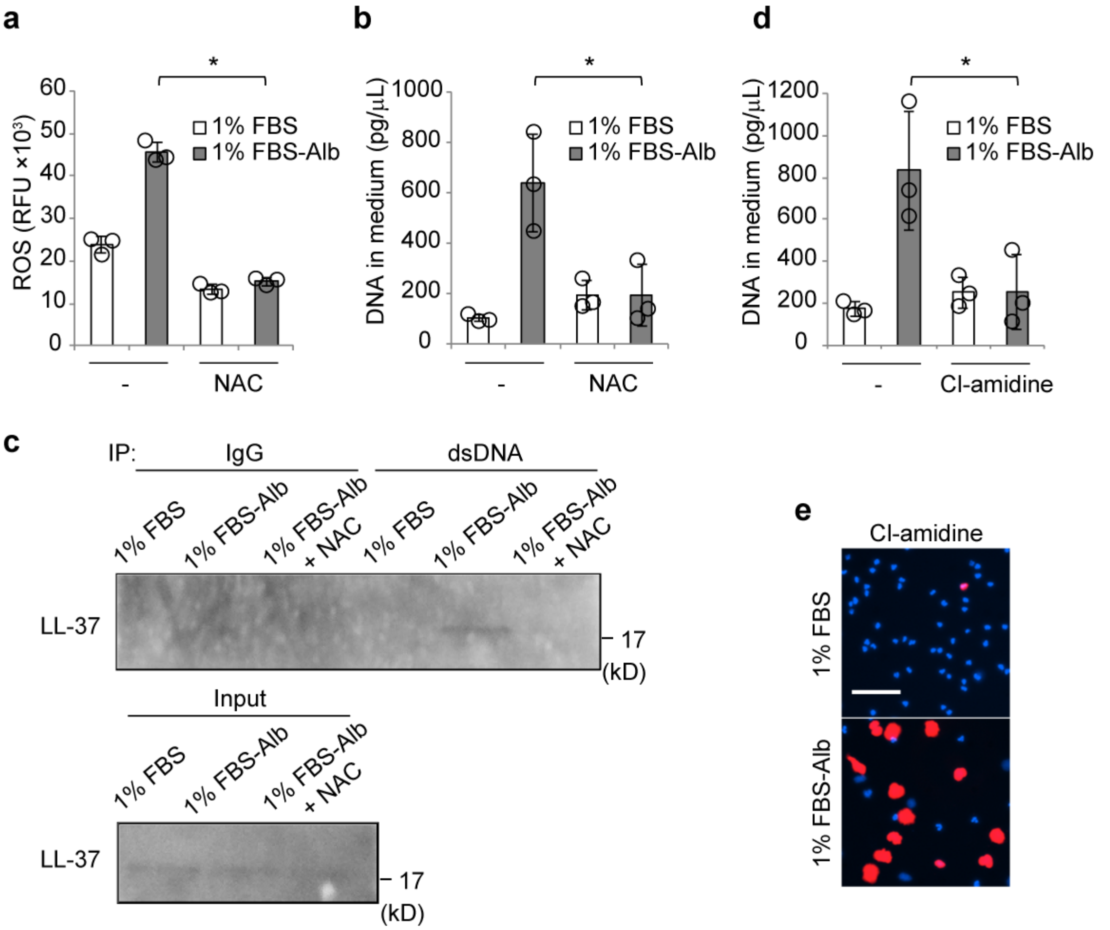

Supplementary Figure 3. PAD inhibition and NAC supplementation prevent albumin depletion-evoked NETosis. (a-d) Human neutrophils were cultured in medium containing 1% FBS or 1% albumin-depleted FBS (1% FBS-Alb) with or without NAC (a, b) or a PAD inhibitor, Cl-amidine (c, d) for 5 minutes (a) and 6 hours (b-d). (a) Intracellular ROS level. (b) Concentration of extracellular DNA within culture medium. (c) Extracellular DNA was immunoprecipitated from culture medium using an anti-dsDNA antibody or IgG isotype control, and the isolated DNA (or the input culture medium without immunoprecipitation) was subjected to Western

blotting for LL-37. (d) Concentration of extracellular DNA within culture medium. (e) Representative images of neutrophils stained with cell-permeable DNA dye, Hoechst 33342 (blue), and cell-impermeable DNA dye, SytoxOrange (red). Bar = 50 μm. (a, b, d) Results represent individual values with the mean ± s.d. (n = 3; biological triplicates. *$P<0.05$ by Student's *t*-test). PAD: peptidyl arginine deiminase. NAC: *N*-acetylcysteine.

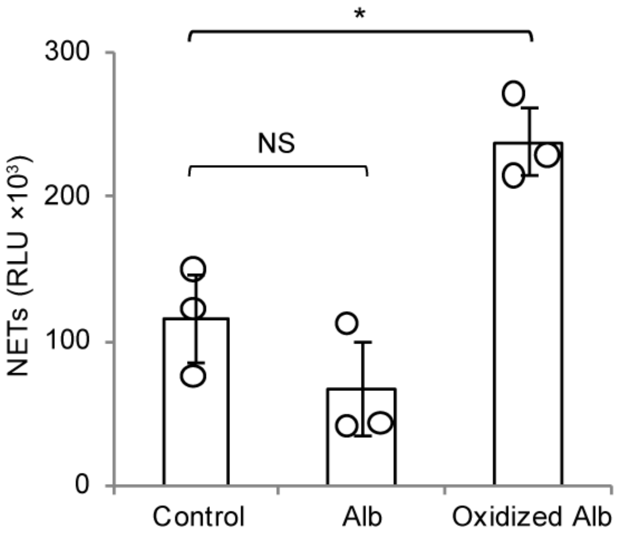

Supplementary Figure 4. Oxidized albumin triggers NETosis within whole blood. Two-milliliter of human whole blood collected in the K2 EDTA tube was incubated with 50 μL of PBS solution of BSA (20 g/dL) or oxidized BSA (20 g/dL) at 37°C for 3 hours with gentle shaking. Isolated plasma was subjected to ELISA to quantify NETs. Results represent individual values with the mean ± s.d. (*n* = 3; technical replicates. NS = not significant. **P*<0.05 by Dunnett's test).

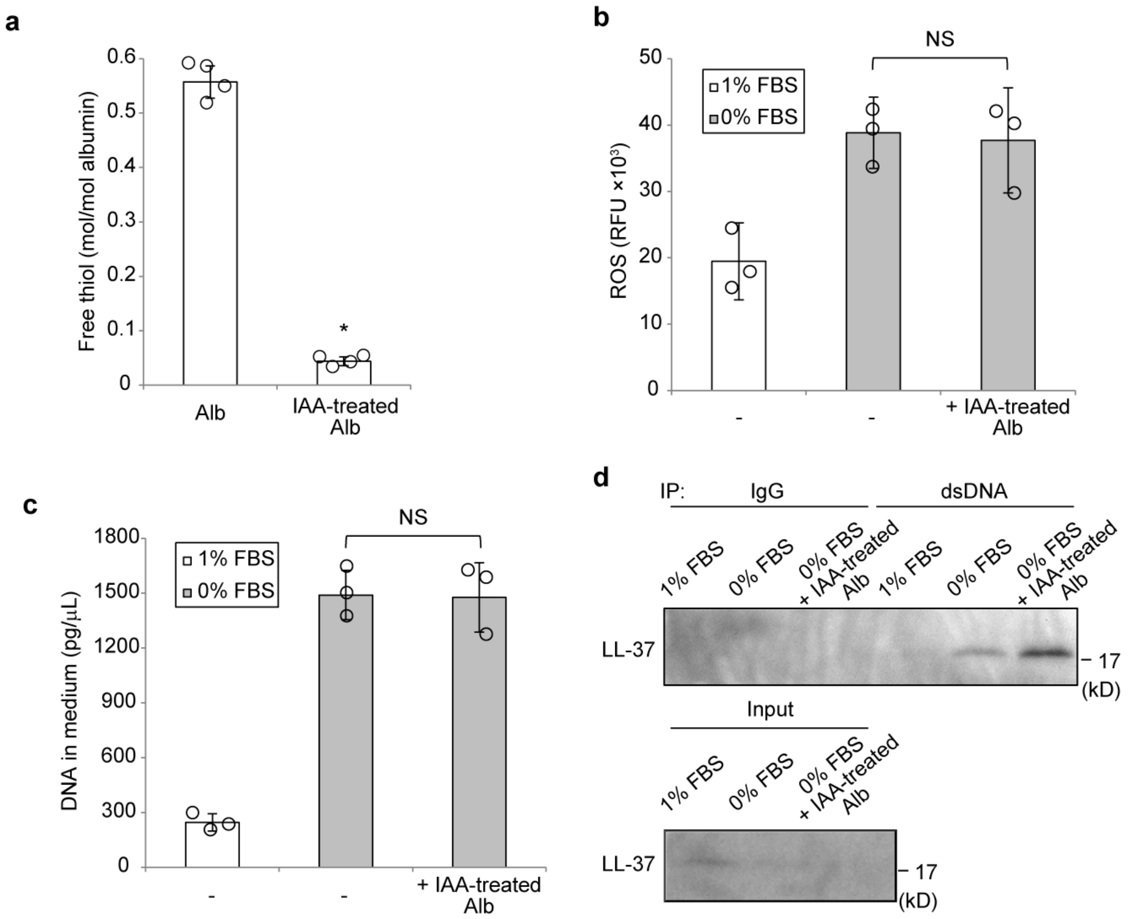

Supplementary Figure 5. Iodoacetamide-treated albumin triggers NETosis through the accumulation of intracellular ROS. (a) Free thiol concentration in the BSA solution with or without free thiol blocking by IAA. (b-d) Neutrophils were cultured in the indicated conditions. (b) Intracellular ROS level was quantified. (c) Concentration of extracellular DNA within culture medium. (d) Extracellular DNA was immunoprecipitated from culture medium using an anti-dsDNA antibody or IgG isotype control, and the isolated DNA (or the input culture medium without immunoprecipitation) was subjected to Western blotting for LL-37. (a-c) Results

represent individual values with the mean ± s.d.. n = 3-4. *P<0.05. NS = not significant by Student's t-test. BSA: bovine serum albumin. ROS: reactive oxygen species. IAA: iodoacetamide.

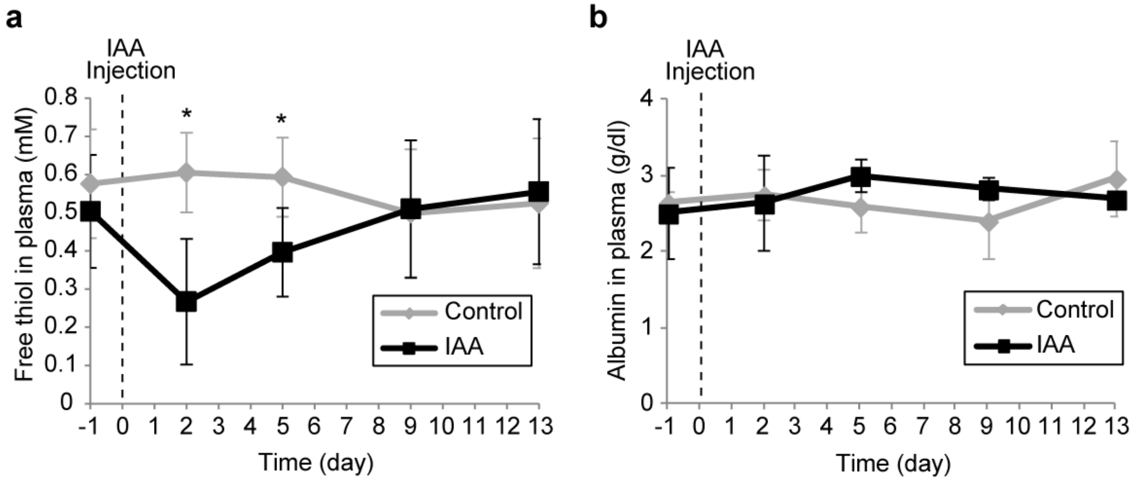

Supplementary Figure 6. Determination of the optimal time point for the detection of NETosis by iodoacetamide. Saline or IAA (30 mg/kg) was injected intraperitoneally into NSG mice. Blood was collected at designated time point. Concentration of plasma free thiol (a) and albumin (b). (a-b) Results represent the mean ± s.d.. *n* = 3. **P*<0.05 by Student's *t*-test at each time point. IAA: iodoacetamide.

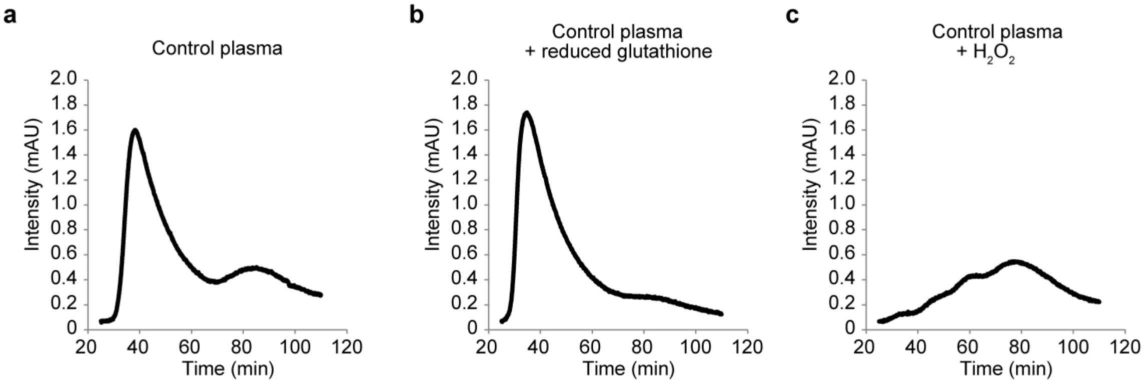

Supplementary Figure 7. Liquid chromatography profile of murine albumin with exogenous reduction and oxidation. Murine albumin in plasma were analyzed by fast protein liquid chromatography. (a) Control mouse plasma. (b and c) Mouse plasma was incubated with 5 mM glutathione for 3 hours (b) or 50 mM hydrogen peroxide for 24 hours (c) to demonstrate that the first and second peaks represent the fraction of reduced and oxidized albumin, respectively.

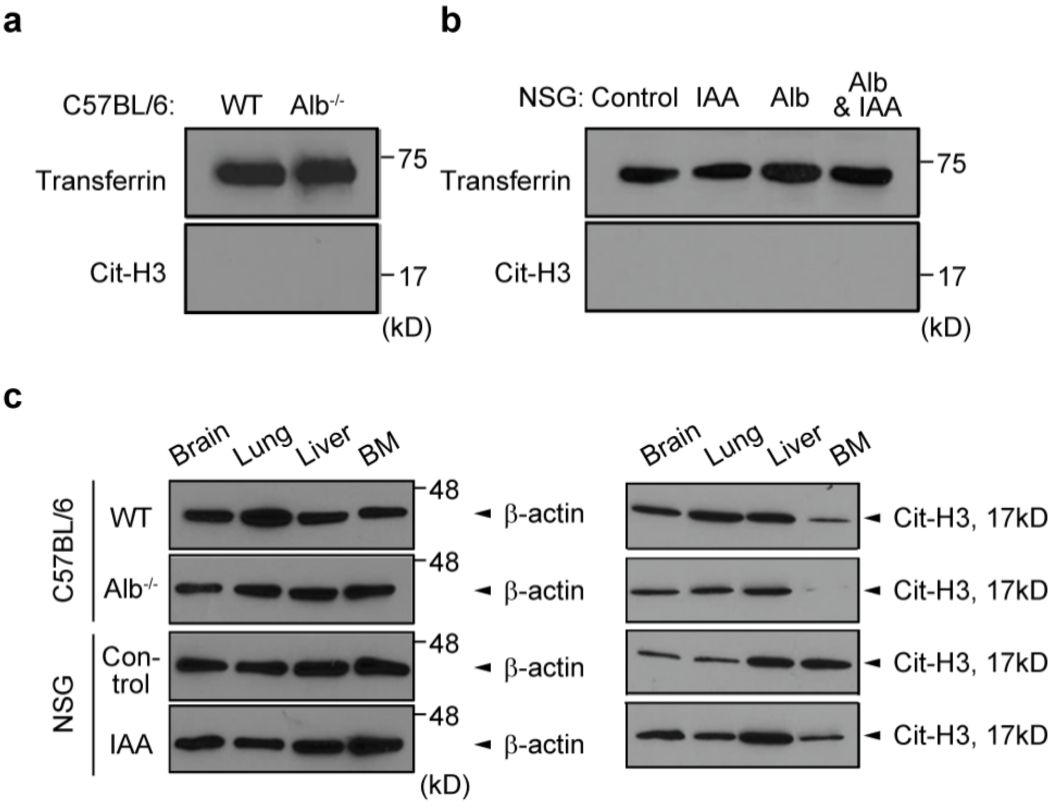

Supplementary Figure 8. Western blot of the loading controls and input controls for immunoprecipitation reactions from plasma and tissue. (a) and (b) As for Figures 2e and 2f, antibodies against transferrin (top) and citrullinated histone H3 (CitH3, bottom) were used for loading control and input control, respectively. (c) As for Figure 3c, antibodies against β-actin (left) and CitH3 (right) were used for loading control and input control, respectively.

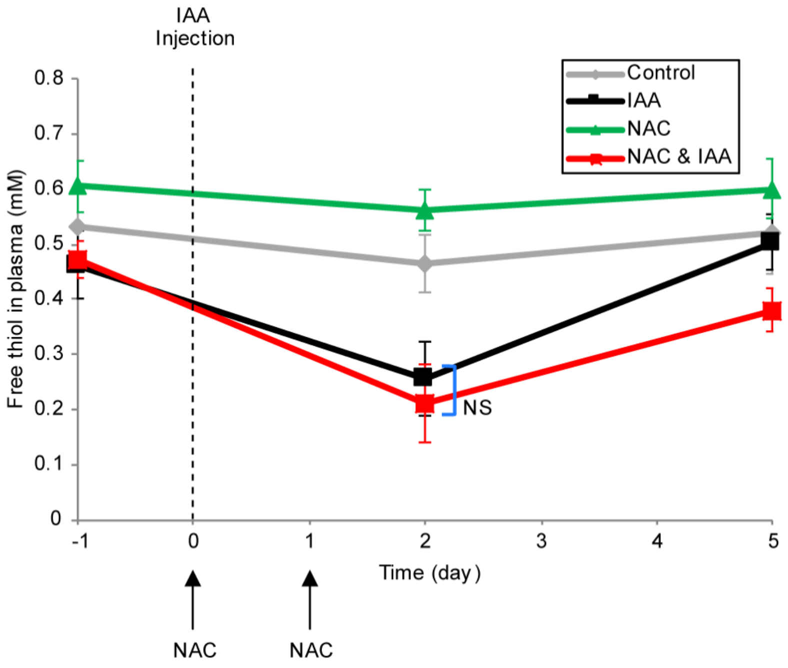

Supplementary Figure 9. NAC is ineffective in normalizing plasma free thiol in IAA-treated mice. Saline solution of NAC (30 mg/ml) was injected intraperitoneally into NSG mice (100 mg/kg) on day 0 and 1. on day 0 saline or IAA (30 mg/kg) was injected intraperitoneally six hours after injection of NAC. Blood was collected at designated time point. Concentration of plasma free thiol was shown. Results represent the mean ± s.d.. n = 3. NS = not significant by Student's t-test. IAA: iodoacetamide. NAC: *N*-acetylcysteine.

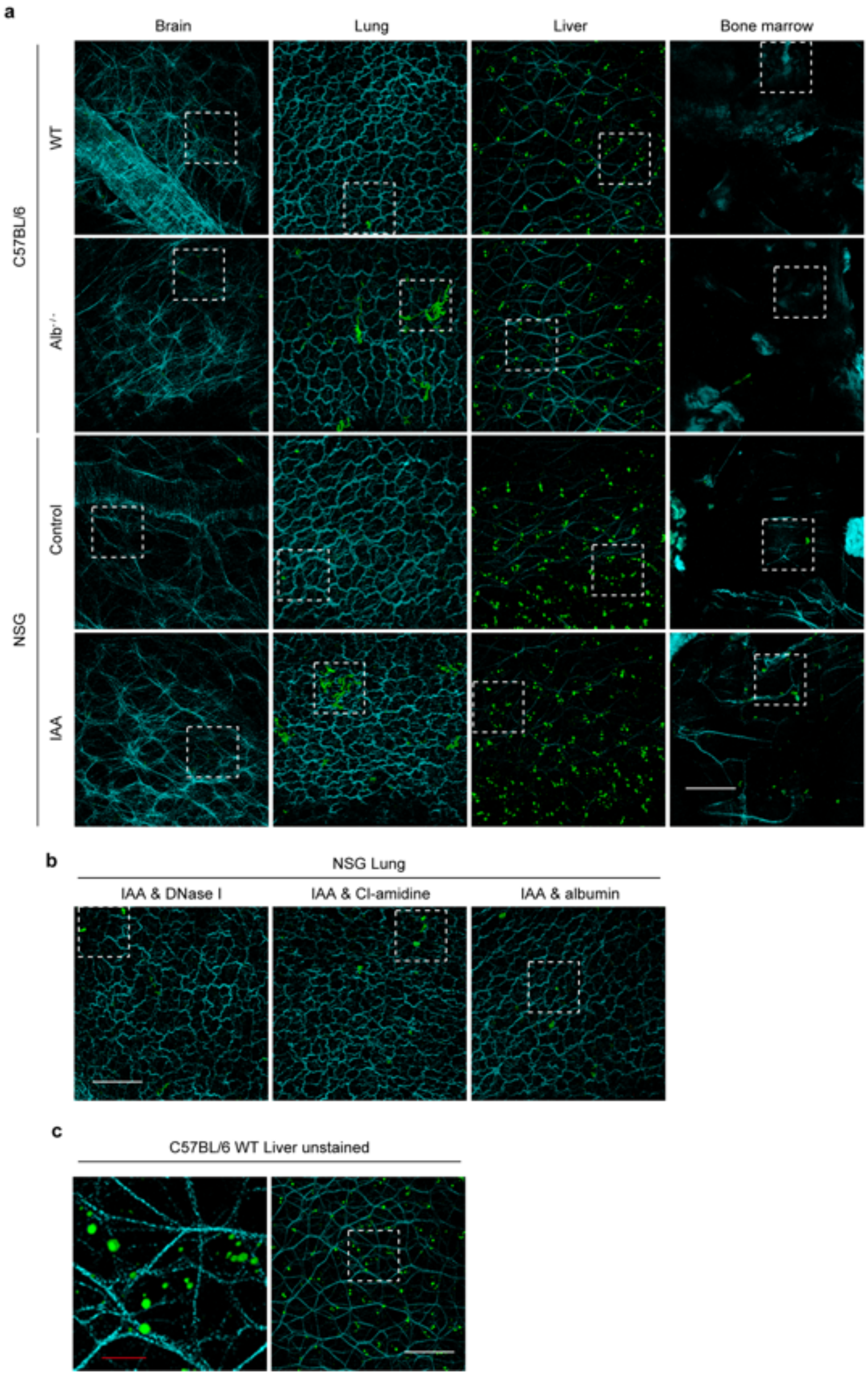
a
Brain
Lung
Liver
Bone marrow
C57BL/6
WT
Alb-/-
NSG
Control
IAA
b
NSG Lung
IAA & DNase I
IAA & Cl-amidine
IAA & albumin
c
C57BL/6 WT Liver unstained

Supplementary Figure 10. Albumin deficiency and albumin thiol-blockade induces lung-predominant NETosis. The indicated organs resected from wild-type (WT) and albumin deficient (Alb$^{-/-}$) C57BL/6 mice and vehicle- and IAA-injected NSG mice were subjected to two-photon microscopy. Surrounding collagen-rich tissues were imaged by second-harmonic generation (shown as cyan). (a, b) To stain extracellular DNA, SytoxGreen was injected *via* tail vein 20 minutes before organ harvest (shown as green). (a) Representative images of brain, lung, liver, and bone marrow. The view in the dotted square is enlarged in Fig. 2h. Bar = 100 μm. (b) In order to inhibit NET formation, single injection of DNase I on day 2, daily injection of Cl-amidine, single injection of murine albumin on day 0 was performed in IAA-injected mice. Representative images of the lung were shown. The view in the dotted square is enlarged in Fig. 2j. Bar = 100 μm (c) Representative image of the unstained liver from WT C57BL/6 untreated mice. The view in the dotted square in the right picture is enlarged in the left. Autofluorescence was shown as green. Red bar = 20 μm; White bar = 100 μm.

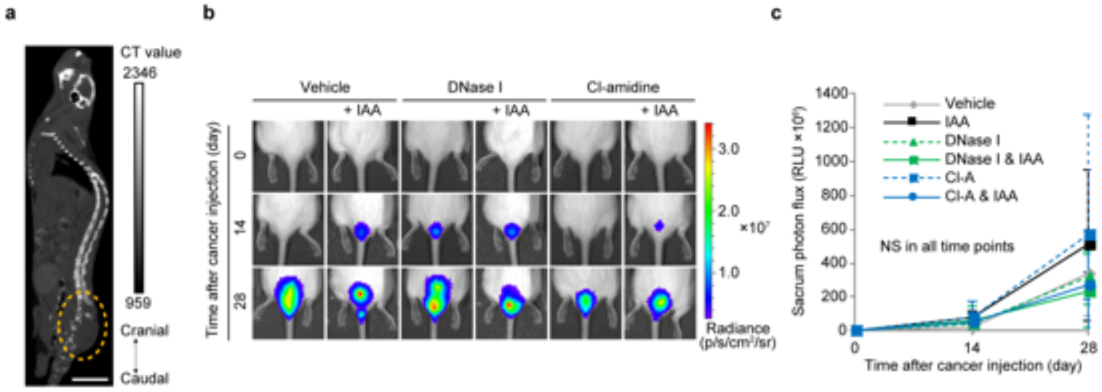

Supplementary Figure 11. NETosis induced by albumin thiol-blockade does not promote bone metastasis. Saline or IAA was injected intraperitoneally into NSG mice. Two day later, CAL-33-luciferase were injected. The growth of sacrum metastases was monitored by bioluminescence imaging. (a) Representative image of computed tomography (CT) imaged 30 days after CAL-33-luciferase injection. The dotted ellipse represents the region of osteolytic sacrum metastasis. Bar = 1.5 cm. (b) Representative bioluminescent images are shown. (c) Luciferase bioluminescence intensity from the sacrum over time (0–28 days). *n* = 5 per group; one of the mice in the DNase I & IAA group died on day 1 for unknown reasons and was excluded. NS = not significant by Dunnett's test.

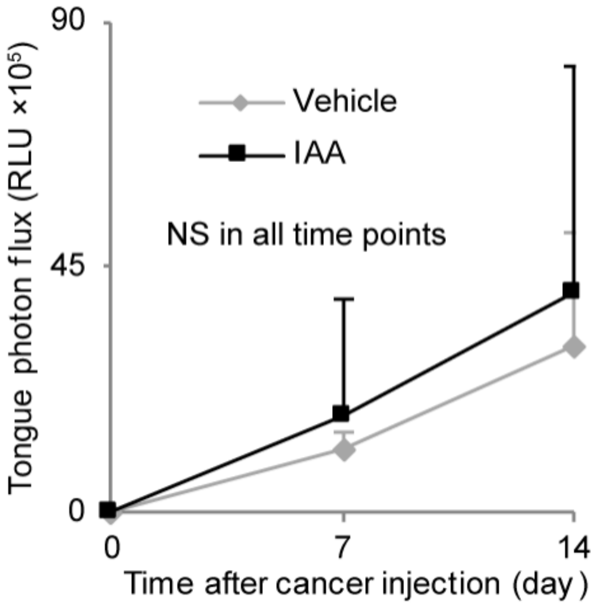

Supplementary Figure 12. Iodoacetamide does not inhibit the growth of CAL-33 tumor. CAL-33-luciferase (5 × 10^4 in 30 μL PBS) was orthotopically injected into the tongue of NSG mice. Three days after tumor implantation, saline or IAA was injected intraperitoneally into mice every three days. The growth of tongue tumor was monitored by bioluminescence imaging (0–14 days). Fifteen days after tumor implantation, mice became unable to intake food due to the tongue tumor and thus were euthanized. *n* = 3 per group. NS = not significant by Student's *t*-test performed in each time point.

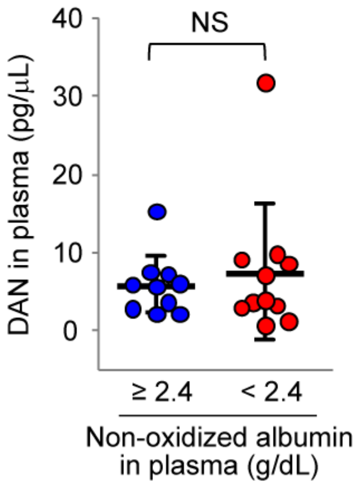

Supplementary Figure 13. Total cell-free DNA concentration for the HNSCC patient cohort. Mid-treatment plasma concentration of cell-free DNA in the HNSCC patient cohort stratified by the median values of non-oxidized albumin. NS = not significant by Student's *t*-test. HNSCC: head and neck squamous cell carcinoma.

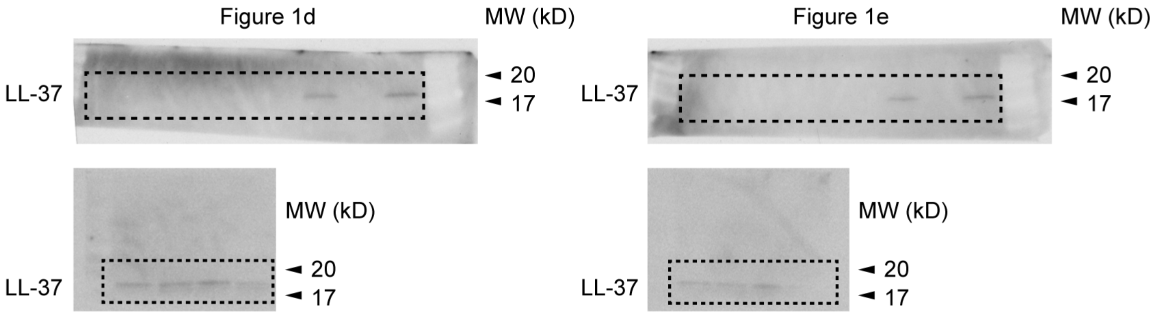

Supplementary Figure 14. Images of the full blots used for the panels shown in Figure 1d and 1e. Boxed areas were cropped for inclusion in indicated figure panels. Proteins and molecular weight markers are indicated on the left and right side of each blot, respectively.

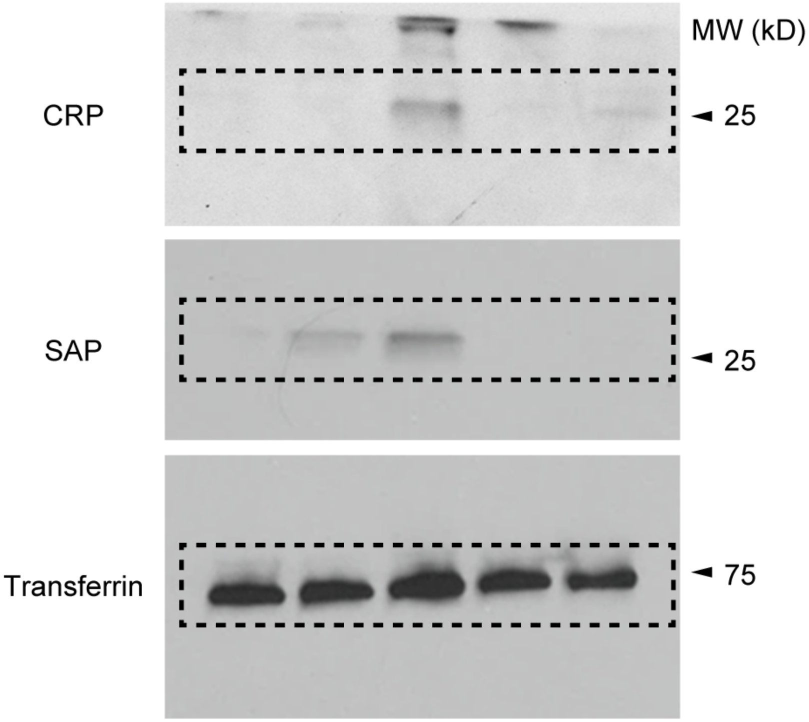

170

Supplementary Figure 15. Images of the full blots used for the panels shown in Figure 2d, 2e and 2f. Boxed areas were cropped for inclusion in indicated figure panels. Proteins and molecular weight markers are indicated on the left and right side of each blot, respectively.

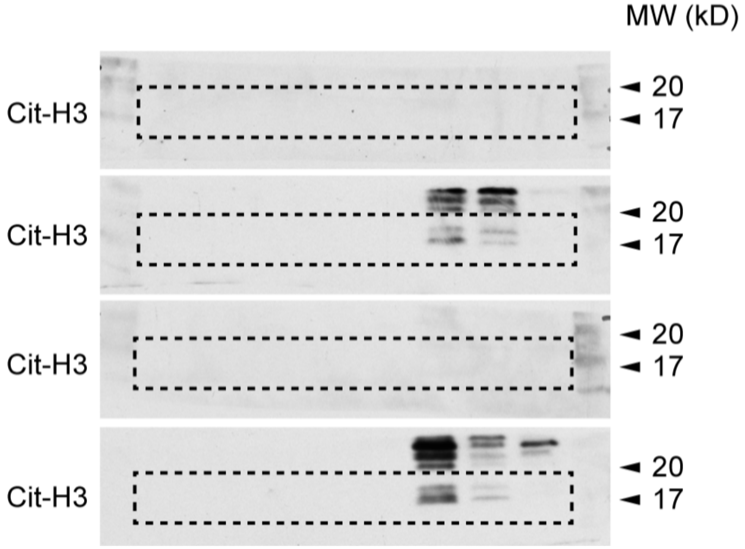

Supplementary Figure 16. Images of the full blots used for the panels shown in Figure 3c. Boxed areas were cropped for inclusion. Proteins and molecular weight markers are indicated on the left and right side of each blot, respectively.

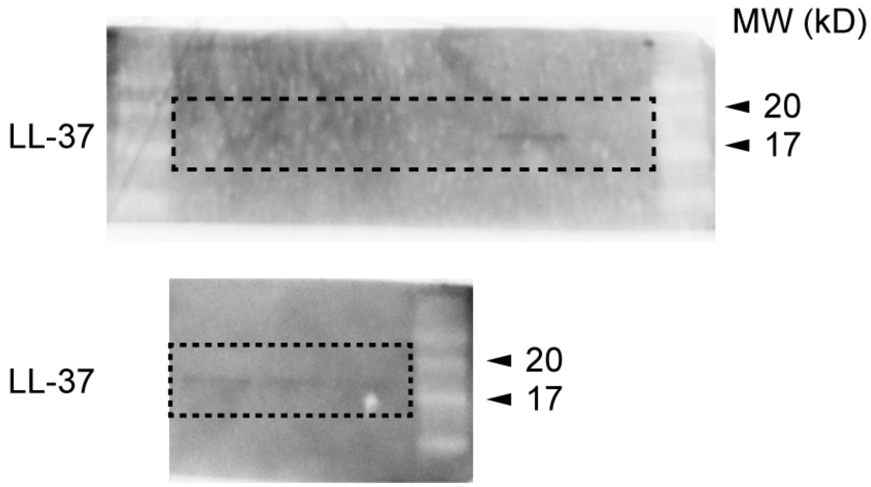

180 **Supplementary Figure 17.** Images of the full blots used for the panels shown in Supplementary Figure 3c. Boxed areas were cropped for inclusion. Proteins and molecular weight markers are indicated on the left and right side of each blot, respectively.

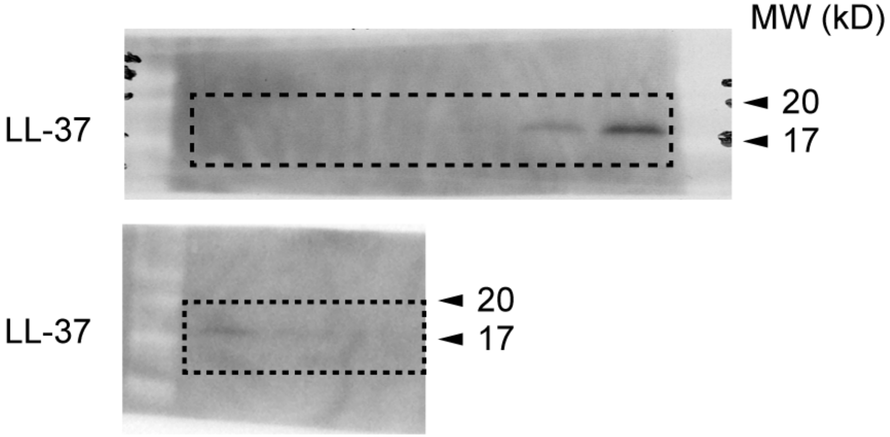

185 **Supplementary Figure 18.** Images of the full blots used for the panels shown in Supplementary Figure 5d. Boxed areas were cropped for inclusion. Proteins and molecular weight markers are indicated on the left and right side of each blot, respectively.

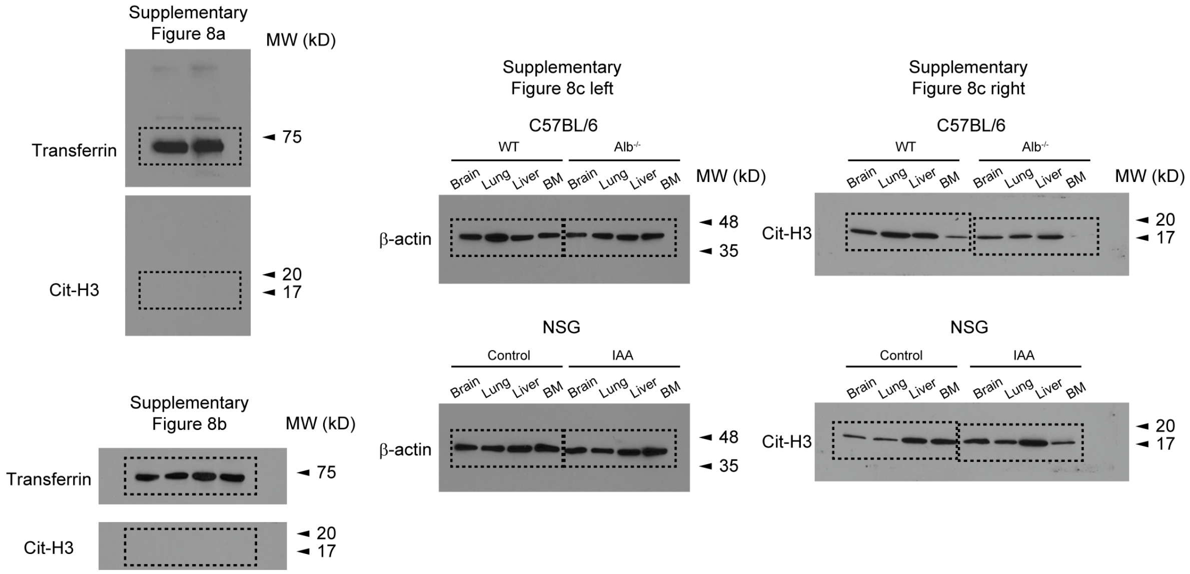

190 **Supplementary Figure 19.** Images of the full blots used for the panels shown in Supplementary Figure 8a, 8b, and 8c. Boxed areas were cropped for inclusion in indicated figure panels. Proteins and molecular weight markers are indicated on the left and right side of each blot, respectively.
